# Ring-Electrode AC Plasmonic Nanopore Sensing for DNA Load Characterization of Single Adeno-Associated Viruses

**DOI:** 10.3390/s26123693

**Published:** 2026-06-10

**Authors:** Scott Renkes, Steven J. Gray, Min Jun Kim, George Alexandrakis

**Affiliations:** 1 Bioengineering Department, University of Texas at Arlington, Arlington, TX 76010, USA; 2 Department of Pediatrics, University of Texas Southwestern, Dallas, TX 75390, USA; steven.gray@utsouthwestern.edu; 3 Department of Mechanical Engineering, Southern Methodist University, Dallas, TX 75205, USA; mjkim@lyle.smu.edu

**Keywords:** plasmonic nanopore, AC nanopore sensing, adeno-associated virus, single-particle sensing, ring electrode, frequency-resolved sensing

## Abstract

Reliable quality control of adeno-associated virus (AAV) vectors remains a major bottleneck in gene therapy manufacturing, particularly for resolving subtle differences in genome loading and conformation at the single-particle level. Existing approaches often struggle to distinguish AAV populations with similar mass and charge, such as capsids carrying self-complementary versus single-stranded DNA. Here, we introduce an AC plasmonic nanopore sensing framework for AAV9 characterization. Individual AAV capsids were optically trapped within a plasmonic double-nanohole nanopore and interrogated using multi-frequency AC pulse trains spanning 500 Hz to 100 kHz. To enhance sensitivity to localized particle–field interactions, a nanofabricated Ag/AgCl ring electrode was integrated concentrically with the plasmonic nanopore. Relative to a conventional wire electrode, the ring electrode produced broader and more robust analyte-dependent differences across multiple frequency-dependent parameters, enabling reliable discrimination of empty capsids (AAV_empty_) and genome-loaded capsids carrying either self-complementary (AAV_scDNA_) or single-stranded DNA (AAV_ssDNA_), despite their near-identical genome mass. Concentration titration experiments further demonstrated that the extracted multivariate AC features remained largely concentration-independent over the tested range. Together, these results demonstrate that ring-electrode-enabled AC plasmonic nanopore sensing provides a multidimensional framework for resolving closely related AAV populations and advances plasmonic nanopores toward practical single-particle quality control of gene therapy vectors.

## 1. Introduction

Solid-state nanopores provide a powerful platform for single-particle analysis by transducing molecular properties into measurable changes in ionic conductance during translocation events [1]. In conventional electrical nanopore sensing, complex particle attributes—including size, charge distribution, shape, and mechanical compliance—are effectively projected onto a single electrical observable. While this approach has enabled robust detection and classification across a wide range of analytes, it becomes increasingly limited when particles share similar mass and net charge, leading to overlapping signatures and reduced separability [2]. This limitation can be understood as a consequence of dimensional compression, where high-dimensional molecular information is mapped onto a low-dimensional measurement space. As the number of distinguishable particle states increases, projection onto a single or a few dimensions inevitably results in information loss, even when underlying physical differences remain substantial.

Plasmonic nanopores can help mitigate this challenge by enabling higher-dimensional measurements (optical and electrical) compared to measurements by solid-state nanopores (electrical only). Specifically, optical trapping and plasmon-enhanced detection provide access to changes in optical transmission, reflectance, and event duration, yielding multiple observables that encode particle size and particle–field interactions [3] that are not accessible by electrical-only measurements. When nanoparticles are the analytes of interest, recent work has demonstrated that combining optical transmission, reflectance, and temporal features significantly expands the effective dimensionality of measurement space [4]. However, these observables still represent a dimensional projection of the underlying molecular state, and physically distinct analytes may remain compressed into overlapping regions of feature space.

To further reduce information loss arising from the dimensional projection, we sought to expand the measurement space while preserving the underlying plasmonic nanopore sensing framework. AC solid-state nanopore sensing has also been proposed recently to enable single-molecule impedance spectroscopy to offer additional measurement dimensions to improve the robustness of analyte characterization by introducing frequency-dependent electrical measurements [5]. However, this work also discussed additional challenges that come with AC measurements: the increased electrical noise relative to DC measurements, combined with the short nanopore translocation times (tens of µs), makes these measurements rather difficult. Our group has also performed prior pilot work on AC sensing using a plasmonic nanopore platform [6]. In that work, the plasmonic nanopore platform consisted of a double nanohole (DNH) plasmonic trap embedded in a gold (Au) layer with a dielectric nanopore milled through an underlying low-stress non-stoichiometric silicon nitride (Si_x_N_y_) layer located at the center of the trap. The Au layer serves as the metallic film from which the DNH plasmonic antenna is formed, enabling optical trapping and detection of analytes at the nanopore. In contrast to the prior work on AC dielectric nanopore sensing [5], we found that AC sensing with a plasmonic nanopore enabled high signal-to-noise ratio (SNR) measurements of protein analytes [6]. This was because the AC electrical measurements were dominated by the free electron response of the plasmonic DNH milled in the Au layer, with the AC signatures being sensitive to the presence of analytes inside the optical trap at the DNH center [6].

Building on these recent developments, the present work extends the application of AC plasmonic nanopore sensing to single nanoparticle characterization and, specifically, the characterization of DNA load heterogeneity in single Adeno-Associated Viruses (AAVs). The interest in AAVs emerges from the fact that they are the leading delivery vectors for in vivo gene therapy, yet reliable quality control (QC) of AAV formulations remains challenging due to the intrinsic heterogeneity of their biomanufactured formulations, leading to potential treatment safety risks [7]. Typical AAV samples contain mixtures of empty capsids (AAV_empty_), partially DNA-filled capsids (AAV_partial_), and fully DNA-loaded capsids (AAV_full_), with only the AAV_full_ population fraction contributing to effective treatment, while AAV_partial_ and AAV_empty_ potentially contribute to immunogenic burden without therapeutic benefit [8]. Furthermore, depending on the therapeutic load, the DNA conformation inside an AAV particle could differ [9].

In this work, this DNA conformation aspect, approximated by the use of AAVs loaded with self-complementary DNA (AAV_scDNA_) or single-stranded DNA (AAV_ssDNA_) of equal base sequence length, is probed by alternating current (AC) plasmonic nanopore sensing. We recently demonstrated that electrical and optical plasmonic nanopore sensing, integrating optical trapping with direct current (DC) measurements, enables label-free, single-particle discrimination between AAVs with different DNA load status and conformation by exploiting differences in capsid deformation, dielectric response, and charge distribution [4]. In that work, clustering-based analysis revealed distinct DNA load type clusters, but overlap between cluster boundaries was observed. The total cluster number was empirically determined with guidance from clustering stability analysis, introducing some degree of subjectivity. Furthermore, in our prior work involving COMSOL Multiphysics (version 5.6, COMSOL, Natick, MA, USA) multi-physics simulations of our plasmonic nanopore sensor [10], we found that certain AC modulation frequencies drive the nanopore current to travel predominantly through the center of an optically trapped nanoparticle. Because the principal physical difference between AAV_scDNA_ and AAV_ssDNA_ resides within the capsid interior, including the presence, organization and conformation of the packaged DNA, these populations are expected to exhibit different internal dielectric properties, charge distributions and charge-relaxation behaviors. AC interrogation therefore provides a means of probing analyte-dependent electrical properties that are directly linked to the DNA load rather than primarily to the capsid geometry alone. These observations motivated the need for developing a frequency-resolved AC interrogation framework to enable more discriminative probing of the DNA-loaded center of single AAV particles to test if this approach can help distinguish between the AAV_empty_, AAV_scDNA_ and AAV_ssDNA_ populations.

An additional challenge observed in prior pilot studies of plasmonic AC sensing was a concentration dependence in the measured signals [6]. In that work, AC signals were collected with Ag/AgCl wire electrodes, resulting in a larger effective sensing volume. Because the electrical response is frequency-dependent, higher-frequency components of the current can couple through dielectric regions not shielded by the Au layer, including the Si_x_N_y_ membrane within the DNH optical trap. To mitigate this effect, a new Ag/AgCl ring electrode was nanofabricated on top of the DNH optical trap to better confine the electrical sensing volume and reduce potential concentration-dependent contributions to the AC measurement. Unlike the wire electrode configuration, the ring electrode geometry was expected to concentrate electric flux near the nanopore center, increasing the fraction of field lines that interact with particles occupying the optical trap while reducing sensitivity to particles located further from the trapping region.

Taken together, the advancements described in this work establish ring-electrode-enabled AC plasmonic nanopore measurements as a multidimensional, frequency-resolved electrical sensing framework that enables discrimination between analytes with closely related physical properties.

## 2. Methods

### 2.1. Sensor Fabrication

Fabrication of the unreleased Si_x_N_y_ membrane stack for the plasmonic nanopore device was performed following previously reported procedures described in Renkes et al. [4]. Devices were fabricated on 4-inch ⟨100⟩ silicon wafers. The baseline device stack consisted of Si_x_N_y_/SiO_2_/Si/SiO_2_/Si_x_N_y_, with nominal layer thicknesses of 60–80 nm Si_x_N_y_, 500 nm SiO_2_, 525 ± 25 µm Si, 500 nm SiO_2_, and 60–80 nm Si_x_N_y_, respectively. The SiO_2_ layers provided electrical insulation, while the thin Si_x_N_y_ layers formed the chemically resistant nanopore membrane. In the present work, devices employing wire electrodes followed the fabrication steps reported in Renkes et al. (2025) [4] without modification. An integrated ring electrode-based sensor was fabricated to enhance AC interrogation using the process described below.

Following completion of the baseline stack, a Au layer liftoff process was performed on what would subsequently serve as the front side of the device. To define the Au layer, a liftoff resist stack was prepared by first applying P20 adhesion promoter at 3000 rpm for 45 s, followed by spin-coating NFR photoresist (JSR Micro, Inc., Sunnyvale, CA, USA) at 3000 rpm for 45 s and soft-baking at 90 °C for 90 s. The resist was patterned using contact photolithography on a SUSS Microtech MA6/BA6 Gen3 contact mask aligner (Suss Microtec, Garching, Germany), followed by a post-exposure bake at 115 °C for 90 s, development in CD-26 developer (MicroChem Corp., Westborough, MA, USA), and an oxygen plasma descum performed in a TePla plasma system (PVA TePla AG, Wettenberg, Hesse, Germany). Metal deposition was performed by electron-beam evaporation using a Thermionics VE-240 system (Thermionics Vacuum Products, Hayward, CA, USA), consisting of a 5 nm Cr adhesion layer followed by a 100 nm Au layer. Liftoff was carried out in acetone under sonication and rinsed with isopropyl alcohol (IPA).

A 100 nm SiO_2_ insulating layer was then deposited on the front side of the device by RF sputtering using an ATC-2400 sputtering system (AJA International, Scituate, MA, USA). To define oxide openings for subsequent nanopore fabrication, a positive photoresist (Shipley S1818) was applied by first spin-coating P20 adhesion promoter at 3000 rpm for 45 s, followed by S1818 at 3000 rpm for 45 s and a pre-exposure bake at 150 °C for 60 s. The resist was patterned by contact photolithography and developed in CD-26 developer. An oxygen plasma descum was performed prior to etching. A window was etched through the SiO_2_ using a 6:1 buffered hydrofluoric acid (BHF) wet etch, exposing the underlying Si_x_N_y_ to enable later nanohole milling. Following the BHF etch, the remaining photoresist was stripped using acetone and rinsed with IPA. This SiO_2_ layer electrically isolated the plasmonic Au layer from subsequently deposited AC electrodes.

Following oxide patterning, the front-side Ag ring electrode (AC electrode) was fabricated using a liftoff process. The same P20/NFR liftoff resist recipe described above was used. The resist was patterned by backside-aligned contact photolithography, post-exposure baked, developed in CD-26 developer, and oxygen plasma descummed prior to metal deposition. Ag was deposited by electron-beam evaporation using the Thermionics VE-240 system, consisting of a 5 nm Cr adhesion layer followed by a 150 nm Ag layer. Liftoff was performed in N-methyl-2-pyrrolidone (NMP) under sonication, followed by acetone and IPA rinses.

Once Ag was introduced to the device, all subsequent cleaning and de-scumming steps were performed exclusively using hydrogen plasma in an Oxford PlasmaLab System 100 reactive ion etcher (RIE) (Oxford Instruments Plasma Technology, Bristol, UK) to prevent Ag oxidation. The wafer was then flipped, and a back-side Ag electrode was fabricated using the same liftoff resist recipe, deposition stack, and liftoff procedure as the front-side Ag ring electrode, with backside alignment used during photolithography and hydrogen plasma descumming performed prior to metal deposition. Following the second Ag liftoff, a positive S1818 photoresist was applied using the same P20/S1818 spin and bake conditions described above and patterned by backside-aligned contact photolithography to define regions for Si_x_N_y_ plasma etching. The etch was performed using the Oxford PlasmaLab System 100 RIE, removing approximately 60–80 nm of Si_x_N_y_ and 500 nm of SiO_2_ to open the membrane window. Immediately following the Si_x_N_y_ etch, the photoresist was removed using a hydrogen plasma clean. The patterned Si_x_N_y_ layer served as a hard mask for subsequent wet etching.

Prior to wet etching, a PMMA protective coating was applied to the front side of the device by first spin-coating P20 adhesion promoter at 3000 rpm for 45 s, followed by PMMA 950K A5 at 2000 rpm for 45 s, and baking at 180 °C for 10 min. Freestanding membranes were then formed by anisotropic silicon etching in KOH, performed using a PEEK wafer holder, following the same etch conditions described in Renkes et al. [4]. After KOH etching, the PMMA protective layer was stripped using acetone and rinsed with IPA. A final hydrogen plasma clean was then performed to remove residual polymer contamination and minimize surface oxidation.

Lastly, nanopores and plasmonic nanostructures, including double nanohole antennas, were milled using a helium ion microscope (ZEISS, Oberkochen, Baden-Württemberg, Germany), following previously reported procedures described in Renkes et al. [4]. Both wire and ring-electrode devices were patterned using identical nanostructure definitions to enable direct comparison between electrode geometries. Photomasks used for all photolithographic patterning were designed in-house and fabricated at Oak Ridge National Laboratory using a DWL-66 laser mask writer (Heidelberg Instruments, Heidelberg, Baden-Württemberg, Germany). Structural characterization of fabricated devices was performed using a ZEISS Merlin field-emission scanning electron microscope (FE-SEM) (ZEISS, Oberkochen, Baden-Württemberg, Germany).

### 2.2. Flow Cell Fabrication and Sample Preparation

Two flow cell configurations were used, corresponding to the wire and ring electrode geometries. The wire electrode flow cell was assembled following previously reported procedures [4]. The ring electrode flow cell was fabricated using a stacked adhesive spacer approach. The fluidic chamber was defined using 12 layers (up to 15 layers in some devices) of SecureSeal™ Adhesive Sheet (SAS-2L) (Grace Bio-Labs, Bend, OR, USA), each with a thickness of 240 µm, stacked onto a PELCO^®^ glass microscope slide (Ted Pella Inc., Redding, CA, USA). The assembly was held in a custom 3D-printed ABS caddy. Electrical leads were attached to silver contact pads using PELCO^®^ conductive carbon adhesive, which was cured in a convective oven at 65 °C for 30 min. After sample loading and sealing, the chip was placed onto the exposed tape and gently pressed to form a watertight seal. Additional adhesive spacer layers (SAS-2L) were positioned as risers to support the coverslip.

Non-therapeutic AAV9 capsids loaded with different forms of the green fluorescent protein (GFP) gene were used as a model system for probing AAV DNA load heterogeneity. Self-complementary (AAV_scDNA_) and single-stranded (AAV_ssDNA_) vectors were produced separately by the University of Texas Southwestern (UTSW) AAV Core, each packaging a GFP expression cassette as described previously [4], with further details in Section 2.4. The two vector constructs differed only in their inverted terminal repeat (ITR) configuration and the presence of a stuffer sequence, resulting in particles with nearly identical total genome mass but distinct internal DNA conformations. This design provides a stringent test case for discriminating AAVs with identical mass but different genome organization. DNA load status and sample integrity were validated against UTSW reference standards using analytical ultracentrifugation and gel electrophoresis, and detailed characterization of these same AAV9 preparations has been reported previously [4]. Samples of AAV_empty_, AAV_ssDNA_, and AAV_scDNA_ were supplied at nanomolar concentrations and serially diluted to 10 fM in 300 mM KCl prior to measurement. For each sample studied, the sensor was loaded with 50 µL of sample solution, and a 170 µm thick glass coverslip was placed on top to enclose the fluidic chamber.

### 2.3. Optical and Electrical Data Acquisition and Analysis

**Figure 1:**
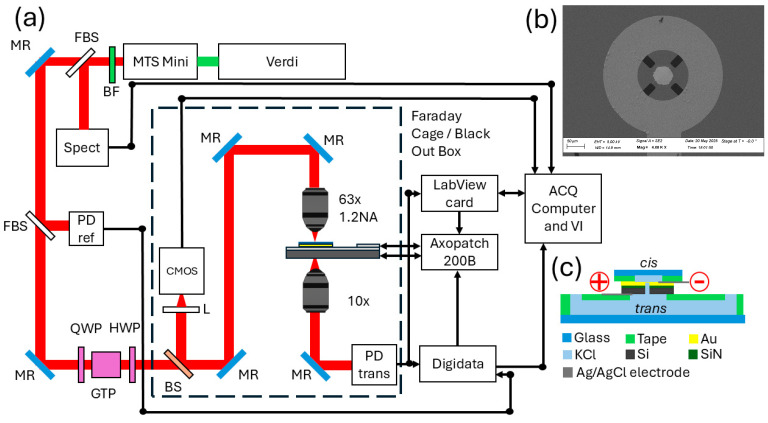
Experimental Setup. (**a**) Schematic of the combined optical and electrical experimental setup. Fused silica beam sampler (FBS), near-infrared mirror (MR), Glan–Thompson polarizer (GTP), half-wave plate (HWP), quarter-wave plate (QWP), 90/10 near-infrared beam splitter (BS), photodiodes (PD), long-pass barrier filter (BF), and camera lens (L). Black arrows represent data channels and the red lines indicate the beam path. (**b**) Scanning electron micrograph (SEM) of the plasmonic nanopore device at 4K× magnification, showing the front-side Ag ring electrode, optical alignment markers, and the oxide window exposing the underlying Au layer into which the double nanohole and nanopore are milled; a corresponding ring electrode is present on the back side of the device. (**c**) Flow cell assembly constructed using layered SAS-2L, defining fluid reservoirs on either side of the membrane for measurements in 300 mM KCl electrolyte.

The optical detection system was based on the plasmonic nanopore platform described previously [4], with modifications implemented to enable real-time triggering of AC electrical pulse trains during optical trapping events. Briefly, a near-infrared excitation beam was focused onto the plasmonic nanopore to enable stable optical trapping, with polarization conditioning and optical alignment performed as described previously [4]. Optical transmission and reflection from the plasmonic nanopore were monitored using photodiodes, and a CMOS camera was used for visual alignment of the excitation beam with the plasmonic DNH. As shown in Figure 1a, the photodetector output was split into two signal paths. One path was routed to a DigiData (Molecular Devices, San Jose, CA, USA) acquisition system for optical recording. The second path was routed to a National Instruments (Austin, TX, USA) PCIe-6376 X-Series DAQ, interfaced through a BNC-2110 terminal block (National Instruments) to act as a trigger for a LabVIEW (2021 SP1) virtual instrument (VI). Electrical recordings were performed using pCLAMP software (11.4.1, Molecular Devices), which served as the master timing reference and primary data recorder. All electrical and optical signals were recorded as ABF files and used for subsequent analysis. The LabVIEW system provided synchronized stimulus generation and control signals to the Axopatch amplifier (Molecular Devices) and auxiliary acquisition hardware. All systems operated in coordination with the pCLAMP acquisition timeline. AC interrogation was applied as a pulse train consisting of multiple discrete frequencies, with each frequency applied for 10 oscillation cycles and separated by short inter-frequency delays. The following frequencies were used: 500 Hz, 1 kHz, 2.5 kHz, 5 kHz, 10 kHz, 25 kHz, 50 kHz and 100 kHz. The magnitudes of the pulses were tuned to maximize the response from the sensor without exceeding 150 nA to prevent triggering the Axopatch overload protection of 200 nA and maximize the command voltage to enable better comparison between the command voltage and signal current. Optical trapping duration was much longer (0.5–20 s) than the pulse train’s total duration (~200 ms), allowing complete multi-frequency AC data acquisition for each AAV particle.

Optical trapping events were identified from step changes in the laser transmission signal. Event detection and tagging were performed using a custom MATLAB script (R2024a). The leading edge of each trapping event was identified, and the first full pulse train was analyzed for each trap. Each analyzed event is treated as a single-particle observation, and the number of analyzed events (n) for each analyte, chip, and electrode configuration is provided in Table S1 of the Supplementary Information.

**Figure 2:**
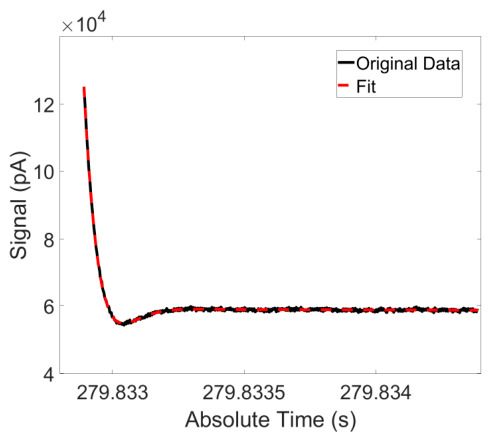
Example of damped oscillation fit (Equation (1)) for ring electrode AAV_ssDNA_ post 1 kHz stimulus termination.

The termination of the 10-cycle of each AC pulse train defined the onset of the post-stimulus transient response. Following our prior work [6], this response was parameterized using a damped oscillator model (Figure 2, Equation (1)), which was selected empirically based on its ability to consistently describe the measured post-AC transient response. A 1.5 ms window following the termination of the AC drive voltage was used for fitting. Fit parameter estimation was performed using nonlinear least-squares optimization with a Levenberg–Marquardt algorithm and robust least absolute residuals (LAR) weighting implemented using MATLAB’s *prepareCurveData()* and *fittype()* functions. Initial parameter values were determined empirically to promote consistent convergence and are noted in Table 1.

(1) $$y(x)=a_{1}+a_{2}x+b_{1}sin\;(c_{1}x+d_{1})\;e^{e_{1}x}.$$

To correct for experimental DC offsets, $a_{1}$ was baseline-corrected using a 1 Hz low-pass filtered version of the signal. Data were windowed around each event, and the mean of the filtered signal within the window was subtracted from the fitted $a_{1}$. Unless otherwise stated, all references to $a_{1}$ in this manuscript refer to the baseline-corrected value. Using 6 fitting parameters for 8 AC frequencies plus the step-change in optical intensity in transmission and reflection, this approach has yielded up to 50 parameters acquired per single AAV particle to use in subsequent analyses.

### 2.4. AAV Vector Preparations

Self-complementary DNA (scAAV9/GFP), single-stranded DNA (ssAAV9/GFP), and empty AAV9 capsids were created and manufactured as described, and the lots used match those in this prior study [11]. In brief, matched sc and ss AAV genomes were created with a total ss DNA length of approximately 4100 nt (or 2050 bp in the self-complementary configuration). These were produced by the UT Southwestern Translational Gene Therapy core by triple transfection of HEK293 cells, followed by cell lysis and filtration, affinity chromatography, and separation of full and empty AAV particles by cesium chloride density gradients.

### 2.5. Statistical Analysis

All statistical analyses were performed in MATLAB (MathWorks, Natick, MA, USA). Statistical testing and feature analysis were designed to determine whether analyte-dependent differences exist, whether these differences are robust relative to intrinsic measurement variability, and whether the resulting feature distributions support discrimination in a classification-relevant sense independent of any specific classifier.

Throughout this work, variability and spread were quantified using the median absolute deviation (MAD), a nonparametric measure of dispersion that is robust to outliers and well suited for small sample sizes [12]. MAD was used both as a direct measure of intrinsic measurement spread and as a normalization factor in subsequent separability and discriminability analyses. The MAD definition used throughout this study is given in Equation (2).

(2) $${MAD(X)=median(|x}_{i}-median(X)|)$$

Initial statistical screening of analyte-dependent differences was performed using pairwise Wilcoxon rank-sum tests implemented with *ranksum()* [13]. This analysis evaluated whether differences between sensor–analyte groupings were statistically detectable without regard to effect size or robustness. Resulting *p*-values were corrected for multiple comparisons using the Benjamini–Hochberg false discovery rate (FDR) procedure implemented with *mafdr()* [14]. Statistical significance was defined as FDR-adjusted *p* < 0.05. Statistical significance throughout the manuscript is denoted using asterisks: *p* < 0.05 (*), *p* < 0.01 (**), and *p* < 0.001 (***), after false discovery rate (FDR) correction. Parameter–frequency pairings were ranked according to corrected *p*-values, and a down-selected subset of the most significant pairings was retained for presentation in the main text, with full statistical matrices provided in the Supporting Information.

Separability was defined as the distance between analyte distributions relative to their intrinsic spread, reflecting how distinctly two analytes are resolved in feature space. This metric is analogous to a z-score, as it expresses the separation between distributions in units of their intrinsic variability but evaluated at the level of distributions rather than individual observations. Separability therefore quantifies whether observed differences reflect meaningful physical distinctions rather than variability arising from finite sampling, independent of any specific decision boundary or classifier. In this work, separability was quantified using a robust standardized distance that is conceptually analogous to standardized effect size metrics such as Cohen’s d and the signal detection index d′ [15,16]. Specifically, the difference between analyte centers was normalized by their pooled intrinsic spread, yielding a variance-normalized measure of distributional separation. To ensure robustness under small-sample and non-Gaussian conditions, medians and median absolute deviations (MADs) were used as nonparametric substitutes for means and standard deviations, respectively [17]. Unlike a classical z-score, this separability metric does not assume normality and is interpreted as a measure of relative distributional separation rather than a probabilistic statistic.

(3) $$S_{robust}=\frac{|median(A)-median(B)|}{\sqrt{\frac{1}{2}({MAD(A)}^{2}+{MAD(B)}^{2})}}$$

Statistical significance of univariate separability metrics was assessed using nonparametric permutation tests [18]. Data from the two analytes were pooled, and group labels were randomly permuted using *randperm()* to generate null distributions. All statistical tests were performed using 5000 random permutations. Empirical two-sided *p*-values were computed as the fraction of permuted statistics greater than or equal to the observed value and corrected using the Benjamini–Hochberg FDR procedure.

Discriminability was defined as the extent to which analyte distributions supported reliable differentiation in a classification-relevant sense, i.e., whether an ideal observer could distinguish analytes based on the measured features between two analyte distributions [16,19]. Univariate discriminability was quantified using an area-under-the-curve (AUC)-based metric, defined in Equation (4) and reported as deviation from chance performance (AUC = 0.5) in Equation (5). AUC represents the probability that a randomly selected observation from one analyte yields a higher discriminant value than a randomly selected observation from another and is mathematically equivalent to the Mann–Whitney U statistic [20]. Statistical significance of discriminability metrics was evaluated using permutation testing analogous to the separability analysis, with FDR correction applied to the resulting *p*-values.

(4) $$AUC=P(X_{A}>X_{B})+\frac{1}{2}P(X_{A}=X_{B})$$

(5) $$S_{AUC}=|AUC-0.5|$$

For visualization of separability in reduced-dimensional feature space, parameter–frequency pairings were ranked by FDR-corrected Wilcoxon significance, and the top three and second top three pairings were selected to generate three-dimensional scatter plots. These plots provided intuitive visual representations of separability identified by the statistical metrics but were not used for quantitative hypothesis testing.

Frequency-dependent trends across analytes were evaluated using the Kruskal–Wallis test implemented with *kruskalwallis()*, with post hoc testing performed using *multcompare()* where appropriate. Boxplots were used to visualize parameter distributions, and trendlines were constructed from group means. Resulting *p*-values were corrected using the Benjamini–Hochberg FDR procedure.

Differences in point spread between two conditions were assessed using a permutation-based robust MAD test, with the observed test statistic defined in Equation (6) [17,18]. Statistical significance was determined by comparing this statistic to a null distribution generated by randomly permuting group labels using *randperm()*, with empirical two-sided *p*-values corrected using the Benjamini–Hochberg FDR procedure.

(6) $$T_{obs}=MAD\left(A\right)-MAD(B)$$

To directly evaluate whether the ring electrode configuration outperformed the wire electrode, separability and discriminability metrics computed for the ring electrode were compared to corresponding values computed for the wire electrode for matched analyte pairings and parameter–frequency bins using permutation-based paired comparisons [18,21]. This analysis tested the null hypothesis that electrode geometry does not affect separability or discriminability.

Finally, to assess separability and discriminability across multiple parameters, observations were represented as multivariate feature vectors. Multivariate separability was quantified using a robust diagonal Mahalanobis-like centroid distance, defined in Equation (7) with pooled scaling defined in Equation (8) [22,23]. Multivariate discriminability was evaluated using a robust linear discriminant score defined in Equation (9), with discriminability quantified via the area under the receiver operating characteristic curve computed from the resulting linear scores [16,20]. Statistical significance of multivariate metrics was assessed using permutation testing with Benjamini–Hochberg FDR correction.

(7) $$S_{multiMAD}={\left\|\frac{m_{A}-m_{B}}{s_{pooled}}\right\|}_{2}$$

(8) $$S_{pooled,k}=\sqrt{\frac{{MAD(A_{k})}^{2}+{MAD(B_{k})}^{2}}{2}}$$

(9) $$w_{k}=\frac{m_{A,k}-m_{B,k}}{s_{pooled,k}^{2}},\;\;\;\;\;\;score=Xw$$

These statistical methods established whether analytes were statistically different, separable relative to intrinsic variability, and discriminable in both univariate and multivariate feature spaces, enabling a classifier-independent comparison of sensing performance based on electrode geometry.

## 3. Results

### 3.1. Pairwise Statistical Differences in Wire Versus Ring Electrode AC Measurements

**Figure 3:**
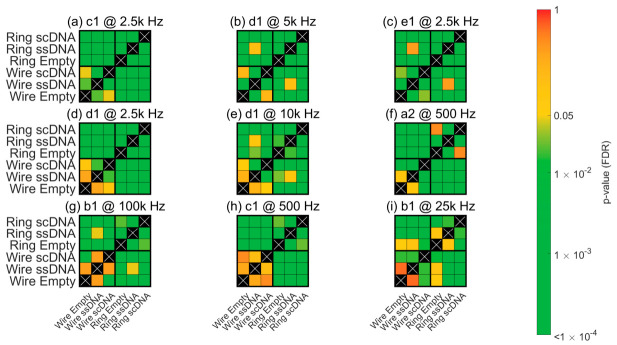
Statistical difference matrices for selected parameter–frequency pairings. Heatmaps of FDR-corrected *p*-values derived from pairwise Wilcoxon rank-sum testing comparing AAV DNA load types across electrode configurations. The nine parameter–frequency combinations shown correspond to the lowest overall adjusted *p*-values across all tested pairings. Color indicates statistical significance, with yellow corresponding to *p* < 0.05 (95% confidence) and green corresponding to *p* < 0.01 (99% confidence).

Pairwise statistical distinguishability of parameter–frequency combinations was evaluated using FDR-corrected *p*-values derived from Wilcoxon rank-sum testing to assess whether analyte-dependent differences were statistically significant across AAV DNA load types and electrode configurations. Sample sizes for each analyte, chip and electrode configuration are provided in Table S1 of the Supplementary Information. To focus on the most informative regions of parameter space, parameter–frequency pairings were ranked according to their adjusted *p*-values, and the nine combinations with the lowest values were selected for further analysis. These down-selected matrices (Figure 3) revealed clear improvements in the ability to statistically differentiate between AAV DNA load types when using sensors with ring electrodes compared to wire electrodes for the same analytes.

For example, the *c*_1_ parameter at 2.5 kHz exhibited statistically significant differences among all analytes for the ring electrode, whereas the wire electrode showed reduced statistical differentiation, particularly between AAV_scDNA_ and AAV_empty_ (Figure 3a). Similar trends were observed for the *d*_1_ (Figure 3d) and *e*_1_ (Figure 3c) parameters, where statistically significant differences are consistently more pronounced when using the ring electrode configuration.

An exception to this general trend was observed for the b_1_ parameter (Figure 3g), where the wire electrode exhibits stronger statistical differentiation between AAV_scDNA_ and AAV_empty_ than the ring electrode.

Taken together, these statistical difference matrices demonstrated that the ring electrode configuration yielded more robust and widespread statistically significant differentiation across the majority of parameter–frequency combinations, motivating subsequent analyses of separability and discriminability based on effect size and robustness.

### 3.2. AAV DNA Load Separability Dependence on the Electrode Type Used for AC Measurements

**Figure 4:**
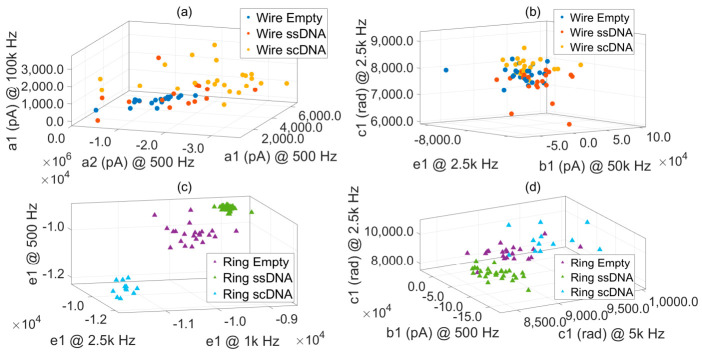
Three-dimensional visualization of statistically informative parameter–frequency combinations. Three-dimensional scatter plots showing reduced-dimensional representations of AAV analyte distributions based on the most statistically informative parameter–frequency pairings identified by FDR-corrected Wilcoxon rank-sum testing. Each point corresponds to a single trapped AAV particle, colored by analyte type. (**a**) Top three parameter–frequency pairings for the wire electrode configuration. (**b**) Second top three parameter–frequency pairings for the wire electrode configuration. (**c**) Top three parameter–frequency pairings for the ring electrode configuration. (**d**) Second top three parameter–frequency pairings for the ring electrode configuration.

To visualize how the use of a wire versus a ring electrode affected the separability of AC measurement-derived model parameters (Equation (1)), scatter plots were generated using the top three and second top three parameter–frequency pairings ranked by FDR-corrected Wilcoxon rank-sum test *p*-values. Figure 4 presents three-dimensional scatter plots for the wire electrode (Figure 4a,b) and the ring electrode (Figure 4c,d). These feature combinations corresponded to the most statistically separable dimensions for each electrode configuration.

For the wire electrode, the top-ranked feature set shows clear separation of AAV_scDNA,_ indicated in Table 2, with separability ranging from 2.04 to 4.26, while AAV_ssDNA_ and AAV_empty_ exhibit partial overlap, indicated by separability values less than 1; larger numbers are a good indicator of separability. The AAV_empty_ population formed a relatively tight grouping, according to Table S2 in the Supplementary Information, with an average spread of −0.08 across the 3 parameters, whereas AAV_scDNA_ displays an increased average spread of 0.68 across the 3 parameters, according to Table S3 in the Supplementary Information. In this case, a negative number indicates a smaller spread than the entire data set, and a positive number indicates a larger spread than the entire data set. In the second-ranked feature set, visual separability is reduced and overlap increases. In contrast, the ring electrode measurements, indicated in Table 3, exhibited improved separability across both feature sets. In the top-ranked pairing, all three analytes formed well-separated clusters with reduced overlap relative to the wire electrode (separability ranging from 3.85 to 37.8). Although the second-ranked feature set shows increased overlap between data clusters, clustering remains more distinct than in the corresponding wire electrode plots (separability ranging from 1.44 to 5.91).

Overall, these visualizations confirm that the statistically selected parameter–frequency pairings correspond to meaningful separation in feature space and that the ring electrode consistently enhanced discrimination between AAV DNA load types.

### 3.3. Frequency-Dependent Trends in Selected AC Parameters

To examine how the separability of AAV DNA load type varied with AC frequency, selected fitted parameters to the damped oscillator model (Equation (1)) were analyzed as a function of AC frequency. The parameters $a_{1}$, $b_{1}$, $d_{1}$, and $e_{1}$ were chosen based on their consistent statistical separability across analytes and electrode configurations.

**Figure 5:**
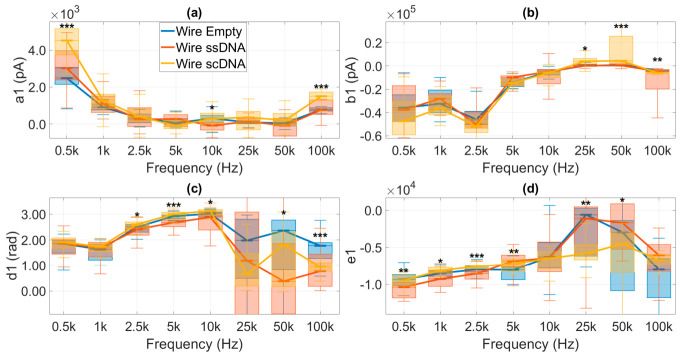
Frequency-dependent distributions of fitted parameters for AAV analytes measured using the wire electrode configuration. Boxplots summarize the distributions at each frequency, with outliers trimmed for visualization. Lines connect group means across frequency to highlight overall trends. Statistical significance between analytes at each frequency was assessed using Kruskal–Wallis testing with FDR correction and is indicated by asterisks. (**a**) a1(f), y-intercept of the damped oscillation. (**b**) b1(f), magnitude of the damped sinusoidal response. (**c**) d1(f), phase delay of the damped oscillation. (**d**) e1(f), exponential decay coefficient. Statistical significance is denoted using asterisks: *p* < 0.05 (*), *p* < 0.01 (**), and *p* < 0.001 (***), after FDR correction.

For the wire electrode (Figure 5), the $a_{1}$ parameter exhibited strong separability at low frequencies (500 Hz), with convergence at intermediate frequencies and renewed divergence between AAV DNA load types at the high-frequency end (100 kHz) (Figure 5a). The $b_{1}$ parameter showed statistically significant separability primarily at 25 kHz and 50 kHz, while increased variability limited significance elsewhere (Figure 5b). The $d_{1}\;$parameter exhibited notable separability in the 2.5–10 kHz range and again at 100 kHz, while variability masked separation at intermediate frequencies (Figure 5c). The $e_{1}$ parameter showed broad-spectrum separability, with AAV_scDNA_ exhibiting a delayed shift relative to the other AAV_ssDNA_ and AAV_Empty_ at higher frequencies (Figure 5d).

**Figure 6:**
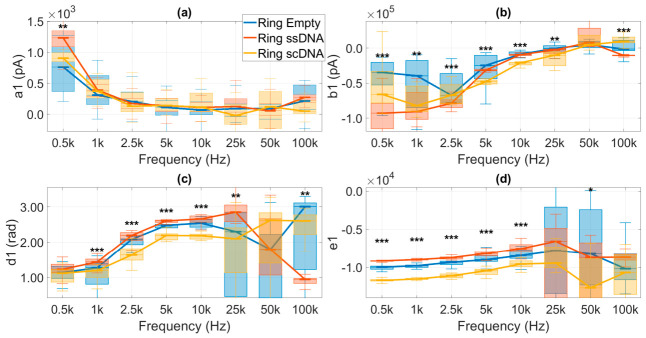
Frequency-dependent parameter distributions for ring electrode measurements. Frequency-dependent distributions of fitted parameters for AAV analytes measured using the ring electrode configuration. Boxplots summarize parameter distributions at each frequency, with outliers trimmed for visualization. Lines connect group means across frequency to highlight overall trends. Statistical significance between analytes at each frequency was assessed using Kruskal–Wallis testing with FDR correction and is indicated by asterisks. (**a**) a1(f), y-intercept of the damped oscillation. (**b**) b1(f), magnitude of the damped sinusoidal response. (**c**) d1(f), phase delay of the damped oscillation. (**d**) e1(f), exponential decay coefficient. Statistical significance is denoted using asterisks: *p* < 0.05 (*), *p* < 0.01 (**), and *p* < 0.001 (***), after FDR correction.

For the ring electrode (Figure 6), separability was enhanced across most parameters and frequencies. The $a_{1}$ parameter retained strong low-frequency separability (Figure 6a), while $b_{1}$ remains separable across most frequencies except 50 kHz, where increased point spread, particularly for AAV_ssDNA_, reduced statistical confidence (Figure 6b). The $d_{1}$ parameter exhibited good separability across most frequencies, with reduced discrimination at 500 Hz and 50 kHz (Figure 6c). The $e_{1}$ parameter showed strong separability up to approximately 10 kHz, with higher-frequency separability increasingly masked by variance (Figure 6d).

Across both electrode configurations, median trends revealed a consistent behavior: the largest separations for $a_{1}$ and $b_{1}$ occurred at low frequencies, $d_{1}$ exhibited frequency-dependent phase shifts with analyte-specific divergence, and $e_{1}$ showed delayed responses for AAV_scDNA_ relative to the other AAV DNA load types.

### 3.4. Multivariate Separability and Discriminability

To evaluate analyte differentiation when considering the joint behavior of multiple frequency-dependent parameters, multivariate separability and discriminability metrics were computed for each analyte pairing and electrode configuration, with results summarized in Table 4. This analysis incorporated all available frequency-dependent dimensions simultaneously, providing a stringent assessment of whether analytes are distinguishable in multidimensional feature space.

For the wire electrode configuration, multivariate performance was limited. Separability values for all analyte pairings were less than one, indicating weak separation relative to intrinsic multivariate spread. DNA-loaded AAV versus AAV_empty_ pairings failed to reject the null hypothesis of no multivariate separability, with only the AAV_ssDNA_ versus AAV_scDNA_ pairing exhibiting separability at the 95% confidence level. Discriminability analysis based on AUC metrics showed comparatively better performance, with AAV_empty_ versus AAV_ssDNA_ identified as discriminable at the 99% confidence level and AAV_ssDNA_ versus AAV_scDNA_ identified as discriminable at a 99.9% confidence level. The other pairings did not reach statistical significance. These results indicate that while certain analyte distinctions may be detectable using wire electrodes, multivariate separability margins are small and therefore susceptible to noise.

In contrast, the ring electrode configuration demonstrated substantially improved multivariate performance. All analyte pairings exhibited multivariate separability values greater than one, with permutation testing indicating statistically significant separation from the null hypothesis of indistinguishable distributions. AUC-based discriminability analysis further showed that all analyte pairings were discriminable, indicating that the combined frequency-dependent feature set supports reliable differentiation in a classification-relevant sense. Together, these results demonstrate that the ring electrode enables robust multidimensional separation of AAV analytes when all available parameters are considered jointly.

### 3.5. AAV Concentration Dependence of Fitted AC Parameters on Electrode Type

To evaluate whether AC-derived multivariate features exhibited concentration dependence, multivariate separability and discriminability analyses were performed for both the wire electrode and the ring electrode configuration across a range of analyte concentrations, with results summarized in Table 5 (wire) and Table 6 (ring). This analysis compared scDNA-containing solutions at three concentrations (10 fM, 1 pM, and 100 pM) against a baseline electrolyte-only condition (300 mM KCl), as well as against one another.

Comparison of the baseline condition to analyte-loaded measurements revealed clear multivariate differentiation for both electrode geometries. For the wire electrode, the baseline was statistically separable from all analyte concentrations, with significant separability observed across the tested concentration range (10 fM, *p* < 0.001; 100 pM, *p* < 0.01). For the ring electrode, the baseline was statistically separable from the 10 fM condition at *p* < 0.05, with statistical significance also observed at higher concentrations (*p* < 0.01 for 100 pM). Multivariate discriminability analysis showed even stronger performance, with all baseline-versus-analyte comparisons yielding *p* < 0.001, indicating robust discriminability between electrolyte-only and analyte-loaded conditions for both electrode configurations.

In contrast, when comparing analyte concentrations to one another, multivariate separability was generally weak, and discriminability did not reach statistical significance. Apart from a single separability comparison in the wire configuration, concentration pairings failed to reject the null hypothesis. Discriminability metrics likewise did not indicate reliable differentiation between concentrations for either electrode geometry.

These results indicate that while both electrode configurations robustly detect the presence of the analyte relative to baseline, neither exhibits strong multivariate differentiation across the tested concentration range. The wire electrode shows slightly greater variability in separability across concentrations, suggesting minor residual concentration sensitivity, whereas the ring electrode response remains more uniformly concentration-independent.

Taken together, these findings demonstrate that the extracted AC multivariate features are largely insensitive to analyte concentration within the examined range. This behavior supports the interpretation that the multivariate AC response is dominated by localized single-particle interactions within the plasmonic trap rather than bulk concentration effects.

## 4. Discussion

This work demonstrates that multi-frequency AC plasmonic nanopore sensing extends electrical nanopore measurements into the frequency domain, providing access to additional, frequency-resolved physical information. A central result is that the incorporation of a nanofabricated ring electrode substantially enhances sensitivity to localized particle-scale dynamics relative to a conventional wire electrode. This improvement arises from a deliberate reduction in electrical probe volume, improved electrode placement reproducibility, and geometry-driven localization of electric flux near the nanopore. Together, these factors shift the measurement regime away from ensemble-averaged bulk electrolyte response and toward single-particle interrogation dominated by AAV-specific properties.

In the wire electrode configuration, a 30-gauge Ag/AgCl wire (~256 µm diameter) was positioned approximately 6.5 mm from the nanopore, producing an extended electric field that sampled a large electrolyte volume and averaged over spatially distributed ionic currents [24]. In contrast, the nanofabricated ring electrode consists of a 150 nm thick silver film with a 100 µm wide lead patterned concentrically around the plasmonic nanopore, with an inner radius of ~70 µm on the front side and ~850 µm on the backside. This geometry placed the active electrode surface two orders of magnitude closer to the nanopore and enforced a non-uniform electric field distribution in which electric field magnitude and current density were preferentially concentrated toward the central axis of the ring [25]. As a result, particles located at or near the nanopore intercept a disproportionately large fraction of the electric flux compared to off-axis particles, suppressing contributions from the surrounding bulk electrolyte and peripheral regions of the flow cell.

The effectiveness of this design strategy is directly validated by the concentration titration experiments performed with the ring electrode. Across a three-order-of-magnitude change in analyte concentration (10 fM, 1 pM, and 100 pM), no statistically significant separability or discriminability was observed between concentrations for the same AAV DNA load type. Quantitatively, multivariate separability values remained below 0.3 with non-significant pAUC (Table S5) across all frequencies, indicating invariance of the fitted response to particle concentration. This absence of concentration dependence confirms that the measured response is not governed by ensemble-averaged bulk impedance but instead reflects single-particle interactions localized to the nanopore region. In conventional distant-electrode configurations, such large concentration changes would be expected to measurably alter admittance through cumulative ionic displacement and distributed particle perturbations [26]. The observed concentration independence therefore provides functional validation that the ring electrode successfully confines the effective sensing volume and enforces flux-weighted interrogation of particles at the nanopore.

Examination of the fitted AC response parameters clarifies which physical features of AAV9 capsids are being probed across frequency and how electrode geometry modulates sensitivity to those features. The constant offset parameter (*a*_1_) behaves as a quasi-static, DC-like component and is most informative at low frequencies, where it reflects slow ion redistribution and access resistance perturbations near the nanopore. These effects depend on genome-dependent ion exclusion and effective capsid charge, which differ between empty and genome-containing capsids [27,28]. Quantitatively, *a*_1_ exhibits consistent low-frequency discrimination under both electrode configurations (AUC ≈ 0.60–0.70 with pAUC < 0.05) (Table S5), but with reduced variability under ring excitation. For example, in the AAV_ssDNA_–AAV_scDNA_ comparison below 1 Hz, *a*_1_ showed a reduction in log-MAD of approximately 0.25 (Table S5) under ring excitation relative to wire excitation, indicating improved single-particle dominance and suppression of bulk contributions.

Importantly, *a*_1_ also provides a practical route to extracting DC-like nanopore information in a measurement regime where direct DC analysis is challenging. In plasmonic nanopore experiments, optical trapping and untrapping events generate large transient electrical spikes that can obscure or mask the steady conductance perturbation associated with a particle’s residence near or within the pore. This makes direct extraction of a translocation current difficult without aggressive temporal filtering [4]. Because *a*_1_ represents the constant offset of the fitted damped response, it captures the quasi-static component of the signal even in the presence of these transient events. As a result, *a*_1_ serves as a proxy for DC-like behavior, measuring the change in baseline conductance levels (off-trap versus on-trap), derived directly from AC data without requiring separate filtering or measurement modes. This unified approach enables simultaneous access to static and dynamic particle properties within a single analytical framework.

Dynamic parameters associated with the oscillatory response—namely the characteristic frequency (*c*_1_), phase offset (*d*_1_), and damping coefficient (*e*_1_)—probe physical mechanisms that dominate at mid-to-high frequencies and are intrinsically surface- and interface-sensitive. These parameters reflect interfacial polarization, electrical double-layer relaxation, and near-surface counterion motion, often described as a skin-current-like contribution [10,26]. Genome loading alters these properties by shifting capsid charge isoforms and redistributing counterions at the capsid–electrolyte interface, even when capsid protein composition remains unchanged [27]. Genome-dependent modulation of capsid surface electrokinetic properties has been independently observed in charge heterogeneity measurements of empty and genome-containing AAV particles [28].

This interpretation is supported quantitatively by univariate analysis. For the damping coefficient (*e*_1_), ring electrode excitation yields substantially stronger differentiation than wire excitation at mid-to-high frequencies. In the AAV_ssDNA_–AAV_scDNA_ comparison at 5–10 kHz, *e*_1_ under ring excitation reaches separability values of approximately 1.2–1.4 with statistically significant pAUC (<0.01) (Table S5), whereas the corresponding wire measurements remain below 0.5 and fail to reach significance. At the same frequencies, *e*_1_ exhibits increased variability under ring excitation, with log-MAD values of approximately 0.5–0.65. The concurrence of increased spread with robust separability indicates heightened sensitivity to physically meaningful dissipation pathways—such as counterion mobility and interfacial loss—rather than increased measurement noise.

The characteristic frequency parameter (*c*_1_) encodes a dominant response timescale associated with charge relaxation and interfacial dynamics. Differences in genome packing density and conformation between self-complementary and single-stranded DNA alter internal charge distribution and effective dielectric response, which in turn modify interfacial relaxation times [25]. Sensitivity of nanopore electrical measurements to nanoparticle deformability and mechanical relaxation has been demonstrated previously using resistive pulse sensing under dynamic conditions [29]. Consistent with this picture, *c*_1_ exhibits moderate but statistically significant separability under ring excitation (separability ≈ 0.8–1.0 with pAUC < 0.05) (Table S5) in frequency bands where wire excitation shows no reliable discrimination, indicating that localized field coupling enhances sensitivity to genome-dependent relaxation dynamics.

The oscillation amplitude parameter (*b*_1_) reflects the strength of coupling between the applied AC field and the particle response and is therefore influenced by particle polarizability, orientation, and position within the optical trap. Under ring excitation, *b*_1_ exhibits increased variability, with log-MAD values approximately twofold higher than under wire excitation at mid-to-high frequencies. Importantly, this increased variability does not eliminate discrimination: separability values for *b*_1_ under ring excitation remain near or above 1.0 with significant pAUC (<0.05) (Table S5) across multiple frequency bins, whereas wire excitation produces both lower MAD and weaker separation. This behavior indicates enhanced sensitivity to spatial and orientational degrees of freedom rather than excess noise, consistent with strong field gradients near the nanopore and prior plasmonic nanopore observations of orientation-dependent coupling [4].

Finally, the selective enhancement of surface- and interface-dominated parameters (*c*_1_, *d*_1_, *e*_1_) under ring excitation suggests sensitivity not only to static genome load state but also to capsid integrity. In aging or degraded AAV preparations, partial genome extrusion, fragmentation, or increased capsid permeability would be expected to alter capsid charge distribution, interfacial polarization, and near-surface ionic dynamics [30]. Because these changes directly affect the same physical mechanisms that dominate the dynamic AC parameters, AC plasmonic nanopore interrogation points toward a potential future application in assessing AAV degradability or genome stability over time, extending single-particle sensing beyond static classification toward functional quality control.

The consistently elevated AUC values observed under ring electrode excitation (Table S5) indicate that the underlying feature space is well-conditioned for downstream classification, even without invoking supervised learning models in the present work.

A practical limitation of the present framework is that the measured feature distributions likely reflect mixtures of multiple underlying subpopulations, such as different capsid fill states and trapping configurations, whose number and relative abundance are not known a priori. Under these conditions, normalization across independently acquired experiments is nontrivial, as conventional scaling approaches risk obscuring physically meaningful distributional structure.

## 5. Conclusions

The results of this work demonstrate that electrode geometry, AC excitation frequency, and parameter selection jointly determine discriminative performance in AC-interrogated nanopore measurements. The nanofabricated ring electrode enhances field localization, suppresses bulk averaging, and amplifies sensitivity to resonance-driven and interfacial dynamics, enabling improved discrimination across multiple fitted parameters relative to a conventional wire electrode. Parameter-specific differences in frequency response and spread further indicate that individual coefficients encode complementary aspects of particle behavior, including quasi-static conductance, dynamic charge relaxation, dissipation, and coupling strength. This multidimensional characterization highlights AC interrogation combined with damped oscillation fitting as a flexible framework for extracting rich molecular information, even in noisy or actively driven plasmonic nanopore environments.

In the present study, sample sizes were sufficiently small that low-abundance subpopulations, such as partially filled or rare AAV capsid states, were unlikely to be observed. Scaling data acquisition will therefore be essential for resolving more complex mixtures containing multiple genome load fractions. At present, direct aggregation of datasets across independently acquired experiments remains limited by the absence of a robust normalization framework. A key challenge is that the measured feature space reflects a mixture of multiple underlying subpopulations, each expected to produce distinct distributional modes (e.g., corresponding to capsid fill states, conformations, or orientations), for which the number, position, and relative weighting are not known a priori. As a result, conventional normalization approaches risk distorting or collapsing physically meaningful structure in the data, preventing consistent alignment of feature distributions between experiments. This limitation is further compounded by relatively low event rates, which restrict the ability to statistically resolve and stabilize these underlying distributions. To address these challenges, future implementations will incorporate flow-based sample exchange to eliminate realignment between analytes and streamline titration measurements, alongside automated alignment and the development of baseline-invariant and distribution-aware normalization strategies. Together, these improvements are expected to enable more consistent data acquisition and support higher-throughput measurements.

## Figures and Tables

**Table 1:** Equation (1) fit parameters and initial conditions.

Parameter	Description	Initial Condition
*a*_1_	Constant offset	Min (signal)
*a*_2_	Linear baseline correction	Min (signal)
*b*_1_	Oscillation amplitude	Mag (signal)
*c*_1_	Natural frequency	4000 rad/s
*d*_1_	Phase offset	1 rad
*e*_1_	Exponential damping coefficient	−0.623

**Table 2:** Wire electrode separability of the top 6 parameter/frequency pairs based on pair-wise comparison *p*-values. Statistical significance is denoted using asterisks: *p* < 0.05 (*), *p* < 0.01 (**), and *p* < 0.001 (***), after FDR correction.

Pairing	*a*_1_ 500 Hz	*a*_1_ 100 kHz	*a*_2_ 500 Hz	*c*_1_ 2.5 kHz	*b*_1_ 50 kHz	*e*_1_ 2.5 kHz
Empty/ssDNA	0.85	0.05	0.76	1.54 *	0.67	1.08 *
Empty/scDNA	3.47 ***	4.26 ***	2.99 ***	0.68	1.82 ***	1.26 *
scDNA/ssDNA	2.04 ***	2.52 ***	2.12 ***	2.30 ***	2.07 ***	2.02 ***

**Table 3:** Ring electrode separability of the top 6 parameter/frequency pairs based on pair-wise comparison *p*-values. Statistical significance is denoted using asterisks: *p* < 0.05 (*), *p* < 0.01 (**), and *p* < 0.001 (***), after FDR correction.

Pairing	*e*_1_ 500 Hz	*e*_1_ 2.5 kHz	*e*_1_ 1 kHz	*c*_1_ 2.5 kHz	*b*_1_ 500 Hz	*c*_1_ 5 kHz
Empty/ssDNA	6.85 ***	3.85 ***	5.72 ***	2.55 ***	3.22 ***	3.57 ***
Empty/scDNA	12.86 ***	7.67 ***	12.22 ***	2.52 ***	1.86 *	2.96 ***
scDNA/ssDNA	29.85 ***	12.43 ***	37.8 ***	5.6 ***	1.44 *	5.91 ***

**Table 4:** Multivariate separability and discriminability of wire and ring electrode AC measurements. Statistical significance is denoted using asterisks: *p* < 0.05 (*), *p* < 0.01 (**), and *p* < 0.001 (***), after FDR correction.

Analyte Pair	Separability Score	Discriminability Score
Wire Empty vs. Wire ssDNA	0.64	0.13 **
Wire Empty vs. Wire scDNA	0.80	0.07
Wire ssDNA vs. Wire scDNA	0.88 *	0.1 ***
Ring Empty vs. Ring ssDNA	1.50 ***	0.14 ***
Ring Empty vs. Ring scDNA	2.26 ***	0.21 ***
Ring ssDNA vs. Ring scDNA	3.60 ***	0.27 ***

**Table 5:** Multivariate separability and discriminability of AC parameters between baseline (no analyte) and AAV_scDNA_ titration measurements in the wire electrode configuration. Statistical significance is denoted using asterisks: *p* < 0.05 (*), *p* < 0.01 (**), and *p* < 0.001 (***), after FDR correction.

Analyte Pair	Separability Score	Discriminability Score
Baseline vs. 100 pM scDNA	2.43 **	0.35 ***
Baseline vs. 10 fM scDNA	3.18 ***	0.40 ***
Baseline vs. 1 pM scDNA	3.31 ***	0.38 ***
10 fM scDNA vs. 100 pM scDNA	1.30	0.06
10 fM scDNA vs. 1 pM scDNA	1.41 *	0.11
1 pM scDNA vs. 100 pM scDNA	0.55	0.08

**Table 6:** Multivariate separability and discriminability of AC parameters between baseline (no analyte) and AAV_scDNA_ titration measurements in the ring electrode configuration. Statistical significance is denoted using asterisks: *p* < 0.05 (*), *p* < 0.01 (**), and *p* < 0.001 (***), after FDR correction.

Analyte Pair	Separability Score	Discriminability Score
Baseline vs. 100 pM scDNA	1.30 **	0.22 ***
Baseline vs. 10 fM scDNA	1.24 *	0.17 ***
Baseline vs. 1 pM scDNA	1.37 **	0.22 ***
10 fM scDNA vs. 100 pM scDNA	0.47	0.04
10 fM scDNA vs. 1 pM scDNA	0.47	0.08
1 pM scDNA vs. 100 pM scDNA	0.50	0.06

## Data Availability

The raw data supporting the conclusions of this article will be made available by the authors on request.

## Supplementary Materials

The following supporting information can be downloaded at: https://www.mdpi.com/article/10.3390/s26123693/s1, Figure S1: Statistical difference matrices for selected parameter–frequency pairings. Heatmaps of FDR-corrected *p*-values derived from pairwise Wilcoxon rank-sum testing comparing AAV DNA load types across electrode configurations. Color indicates statistical significance, with yellow corresponding to *p* < 0.05 (95% confidence) and green corresponding to *p* < 0.01 (99% confidence); Table S1: Number of analyzed events (n) per analyte and electrode configuration. Each event corresponds to a single optical trapping event and is treated as an independent single-particle observation. Event counts are reported for all analyte types (AAVempty, AAVssDNA, AAVscDNA) under both wire and ring electrode configurations; Table S2: Pairwise point spread table for Wire/Ring pair with Empty analytes. A positive logMAD indicates a greater point spread when compared to all experimental data and a negative logMAD indicates less spread; Table S3: Pairwise point spread table for Wire/Ring pair with scDNA analytes. A positive logMAD indicates a greater point spread when compared to all experimental data and a negative logMAD indicates less spread; Table S4: Pairwise point spread table for Wire/Ring pair with ssDNA analytes. A positive logMAD indicates a greater point spread when compared to all experimental data and a negative logMAD indicates less spread; Table S5: Separability and Discriminability for analyte pairings with each electrode.
