# Supplementary material for: Ring-Electrode AC Plasmonic Nanopore Sensing for DNA Load Characterization of Single Adeno-Associated Viruses
Source: Sensors (Basel). 2026 Jun 10;26(12):3693. doi: 10.3390/s26123693 (PMC13306443; doi:10.3390/s26123693)
Supplement: Supplementary file 1 [file sensors-26-03693-s001.zip › sensors-4322836-supplementary.pdf]

Supplementary Information for: Ring-Electrode AC Plasmonic Nanopore Sensing for DNA Load Characterization of Single Adeno-Associated Viruses

Scott Renkes[1,*], Steven J. Gray[2], Min Jun Kim[3] and George Alexandrakis[1,*]

[1] Bioengineering Department, University of Texas at Arlington, Arlington, TX 76010, USA
[2] Department of Pediatrics, University of Texas Southwestern, Dallas, TX 75390, USA; steven.gray@utsouthwestern.edu
[3] Department of Mechanical Engineering, Southern Methodist University, Dallas, TX 75205, USA; mjkim@lyle.smu.edu
[*] Correspondence: scott.renkes@uta.edu (S.R.); galex@uta.edu (G.A.)

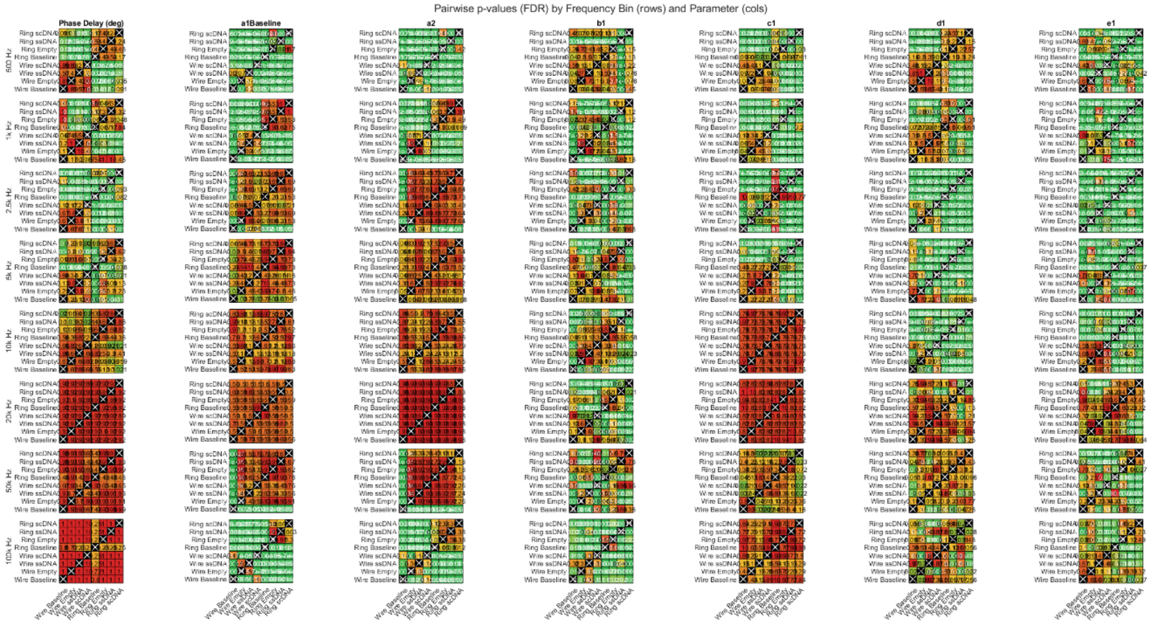

Figure S1: Statistical difference matrices for selected parameter–frequency pairings. Heatmaps of FDR-corrected p-values derived from pairwise Wilcoxon rank-sum testing comparing AAV DNA load types across electrode configurations. Color indicates statistical significance, with yellow corresponding to p < 0.05 (95% confidence) and green corresponding to p < 0.01 (99% confidence).

Table S1: Number of analyzed events (n) per analyte and electrode configuration. Each event corresponds to a single optical trapping event and is treated as an independent single-particle observation. Event counts are reported for all analyte types (AAVempty, AAVssDNA, AAVscDNA) under both wire and ring electrode configurations.

Analyte	Electrode	Chip	Concentration	Events
Baseline	Wire	B3S8-15	N/A	10
AAV_{empty}	Wire	B3S8-5	10 fM	21
AAV_{ssDNA}	Wire	B3S8-5	10 fM	29
AAV_{scDNA}	Wire	B3S8-5	10 fM	12
AAV_{scDNA}	Wire	B3S8-15	10 fM	10
AAV_{scDNA}	Wire	B3S8-15	1 pM	10
AAV_{scDNA}	Wire	B3S8-15	100 pM	10
Baseline	Ring	Ring2	N/A	10
AAV_{empty}	Ring	Ring	10 fM	22
AAV_{ssDNA}	Ring	Ring	10 fM	29
AAV_{scDNA}	Ring	Ring	10 fM	12
AAV_{scDNA}	Ring	Ring2	10 fM	14
AAV_{scDNA}	Ring	Ring2	1 pM	10
AAV_{scDNA}	Ring	Ring2	100 pM	12

Table S2: Pairwise point spread table for Wire/Ring pair with Empty analytes. A positive logMAD indicates a greater point spread when compared to all experimental data and a negative logMAD indicates less spread.

Parameter	Frequency	Wire Empty logMAD	Ring Empty logMAD	p
a1	100000	-0.429	0.429	0.1163
a1	10000	0.317	-0.317	0.0540
a1	1000	-0.104	0.104	0.8037
a1	2500	0.292	-0.292	0.1647
a1	25000	-0.156	0.156	0.5319
a1	500	0.034	-0.034	0.8594
a1	50000	0.187	-0.187	0.4869
a1	5000	0.126	-0.126	0.7776
a1	Total	0.320	-0.320	0.0000
a2	100000	-0.313	0.313	0.3001
a2	10000	0.501	-0.501	0.0302
a2	1000	-0.012	0.012	0.9710
a2	2500	0.268	-0.268	0.2189
a2	25000	0.057	-0.057	0.7013
a2	500	0.154	-0.154	0.4440
a2	50000	0.241	-0.241	0.2366
a2	5000	-0.007	0.007	0.9710
a2	Total	0.316	-0.316	0.0000
b1	100000	-1.499	1.499	0.0198
b1	10000	-0.114	0.114	0.6909

b1	1000	-0.386	0.386	0.0858
b1	2500	-0.458	0.458	0.1787
b1	25000	-1.070	1.070	0.0612
b1	500	-0.137	0.137	0.5247
b1	50000	-0.768	0.768	0.0612
b1	5000	-0.738	0.738	0.0270
b1	Total	-0.193	0.193	0.0858
c1	100000	0.487	-0.487	0.6868
c1	10000	0.382	-0.382	0.0378
c1	1000	-0.126	0.126	0.9728
c1	2500	-0.022	0.022	0.9851
c1	25000	-0.025	0.025	0.9851
c1	500	0.330	-0.330	0.6828
c1	50000	0.162	-0.162	0.0378
c1	5000	0.892	-0.892	0.0156
c1	Total	0.302	-0.302	0.0011
d1	100000	-0.642	0.642	0.0000
d1	10000	-0.127	0.127	0.9322
d1	1000	0.142	-0.142	0.9322
d1	2500	0.268	-0.268	0.9322
d1	25000	0.065	-0.065	0.9322
d1	500	0.313	-0.313	0.6653
d1	50000	0.011	-0.011	0.9322
d1	5000	0.066	-0.066	0.9322
d1	Total	-0.016	0.016	0.9322
e1	100000	0.108	-0.108	0.7264
e1	10000	0.399	-0.399	0.1271
e1	1000	0.374	-0.374	0.4941
e1	2500	0.274	-0.274	0.7264
e1	25000	-0.585	0.585	0.0839
e1	500	0.693	-0.693	0.0011
e1	50000	-0.198	0.198	0.5403
e1	5000	0.165	-0.165	0.5468
e1	Total	0.222	-0.222	0.0155
PhaseDelay	100000	-0.301	0.301	0.9857
PhaseDelay	10000	0.155	-0.155	0.8735
PhaseDelay	1000	0.004	-0.004	0.9924
PhaseDelay	2500	0.193	-0.193	0.7410
PhaseDelay	25000	0.000	0.000	0.8171
PhaseDelay	500	0.165	-0.165	0.7701
PhaseDelay	50000	-0.138	0.138	0.6847
PhaseDelay	5000	0.303	-0.303	0.6847
PhaseDelay	Total	-0.099	0.099	0.5852

Table S3: Pairwise point spread table for Wire/Ring pair with scDNA analytes. A positive logMAD indicates a greater point spread when compared to all experimental data and a negative logMAD indicates less spread.

Parameter	Frequency	Wire scDNA logMAD	Ring scDNA logMAD	p
a1	500	1.069	-1.069	0.6447
a1	1000	0.434	-0.434	0.1954
a1	2500	0.502	-0.502	0.1948
a1	5000	0.469	-0.469	0.1647
a1	10000	0.358	-0.358	0.3205
a1	25000	0.302	-0.302	0.3321
a1	50000	-0.051	0.051	0.8037
a1	100000	0.266	-0.266	0.8301
a1	Total	0.591	-0.591	0.0000
a2	500	0.716	-0.716	0.5614
a2	1000	0.474	-0.474	0.2366
a2	2500	0.231	-0.231	0.3739
a2	5000	0.494	-0.494	0.0827
a2	10000	0.611	-0.611	0.0827
a2	25000	0.463	-0.463	0.0827
a2	50000	0.269	-0.269	0.3634
a2	100000	0.564	-0.564	0.3001
a2	Total	0.436	-0.436	0.0000
b1	500	-0.143	0.143	0.6909
b1	1000	-0.235	0.235	0.5273
b1	2500	-0.389	0.389	0.0856
b1	5000	0.103	-0.103	0.9634
b1	10000	0.372	-0.372	0.9634
b1	25000	-0.298	0.298	0.6985
b1	50000	0.243	-0.243	0.6909
b1	100000	-0.679	0.679	0.6909
b1	Total	-0.332	0.332	0.0000
c1	500	0.063	-0.063	0.9851
c1	1000	0.550	-0.550	0.8901
c1	2500	-0.103	0.103	0.9851
c1	5000	0.511	-0.511	0.1936
c1	10000	1.007	-1.007	0.0806
c1	25000	0.000	0.000	0.9980
c1	50000	-0.426	0.426	0.0155
c1	100000	-0.097	0.097	0.9728
c1	Total	-0.011	0.011	0.9851
d1	500	0.214	-0.214	0.9322
d1	1000	0.259	-0.259	0.9322
d1	2500	-0.151	0.151	0.9322

d1	5000	0.171	-0.171	0.9322
d1	10000	0.144	-0.144	0.9322
d1	25000	0.207	-0.207	0.9322
d1	50000	0.169	-0.169	0.7614
d1	100000	-0.260	0.260	0.9322
d1	Total	0.160	-0.160	0.0410
e1	500	0.820	-0.820	0.1554
e1	1000	0.746	-0.746	0.7264
e1	2500	0.018	-0.018	0.9932
e1	5000	0.464	-0.464	0.4941
e1	10000	0.427	-0.427	0.3956
e1	25000	-0.043	0.043	0.9932
e1	50000	-0.463	0.463	0.0839
e1	100000	-0.562	0.562	0.1271
e1	Total	0.158	-0.158	0.2974
PhaseDelay	500	0.193	-0.193	0.7701
PhaseDelay	1000	-0.303	0.303	0.7410
PhaseDelay	2500	0.062	-0.062	0.9857
PhaseDelay	5000	0.175	-0.175	0.7410
PhaseDelay	10000	0.799	-0.799	0.4338
PhaseDelay	25000	0.658	-0.658	0.4338
PhaseDelay	50000	0.398	-0.398	0.9391
PhaseDelay	100000	0.176	-0.176	0.9486
PhaseDelay	Total	-0.121	0.121	0.4320

Table S4: Pairwise point spread table for Wire/Ring pair with ssDNA analytes. A positive logMAD indicates a greater point spread when compared to all experimental data and a negative logMAD indicates less spread.

Parameter	Frequency	Wire ssDNA logMAD	Ring ssDNA logMAD	p
a1	500	0.789	-0.789	0.1548
a1	1000	0.627	-0.627	0.0077
a1	2500	0.718	-0.718	0.0000
a1	5000	0.429	-0.429	0.0027
a1	10000	0.464	-0.464	0.0128
a1	25000	0.061	-0.061	0.8301
a1	50000	0.651	-0.651	0.0000
a1	100000	0.691	-0.691	0.0330
a1	Total	0.580	-0.580	0.0000
a2	500	0.524	-0.524	0.1909
a2	1000	0.539	-0.539	0.0085
a2	2500	0.802	-0.802	0.0000
a2	5000	0.594	-0.594	0.0000

a2	10000	0.505	-0.505	0.0302
a2	25000	0.029	-0.029	0.9606
a2	50000	0.508	-0.508	0.0036
a2	100000	0.749	-0.749	0.0088
a2	Total	0.483	-0.483	0.0000
b1	500	-0.087	0.087	0.7950
b1	1000	-0.336	0.336	0.5440
b1	2500	-0.028	0.028	0.9634
b1	5000	0.338	-0.338	0.8613
b1	10000	0.344	-0.344	0.7262
b1	25000	-0.506	0.506	0.5273
b1	50000	-0.893	0.893	0.0983
b1	100000	0.482	-0.482	0.8613
b1	Total	-0.488	0.488	0.0000
c1	500	0.978	-0.978	0.0154
c1	1000	1.057	-1.057	0.0000
c1	2500	0.332	-0.332	0.8901
c1	5000	0.910	-0.910	0.0000
c1	10000	0.850	-0.850	0.0000
c1	25000	0.718	-0.718	0.0108
c1	50000	0.213	-0.213	0.4572
c1	100000	0.867	-0.867	0.0238
c1	Total	0.714	-0.714	0.0000
d1	500	0.181	-0.181	0.9322
d1	1000	0.430	-0.430	0.1520
d1	2500	0.202	-0.202	0.7614
d1	5000	0.685	-0.685	0.0338
d1	10000	0.380	-0.380	0.4192
d1	25000	0.897	-0.897	0.0162
d1	50000	0.205	-0.205	0.0837
d1	100000	0.835	-0.835	0.0338
d1	Total	0.059	-0.059	0.6653
e1	500	1.039	-1.039	0.0036
e1	1000	1.257	-1.257	0.0000
e1	2500	0.810	-0.810	0.0000
e1	5000	0.501	-0.501	0.0139
e1	10000	0.659	-0.659	0.0000
e1	25000	0.002	-0.002	0.9932
e1	50000	0.040	-0.040	0.9932
e1	100000	0.611	-0.611	0.0839
e1	Total	0.635	-0.635	0.0000
PhaseDelay	500	-0.207	0.207	0.7701
PhaseDelay	1000	0.035	-0.035	0.9486

PhaseDelay	2500	0.322	-0.322	0.4320
PhaseDelay	5000	-0.054	0.054	0.9486
PhaseDelay	10000	0.206	-0.206	0.7410
PhaseDelay	25000	0.135	-0.135	0.4320
PhaseDelay	50000	0.000	0.000	0.7701
PhaseDelay	100000	8.182	-8.182	0.4338
PhaseDelay	Total	-0.121	0.121	0.5852

Table S5: Separability and Discriminability for analyte pairings with each electrode.

SetA	SetB	Param	Frequency	Separability	p	AUC	pAUC
Ring Empty	Ring ssDNA	a1	500	1.509	0.0008	0.304	0.0182
Ring Empty	Ring ssDNA	a1	1000	0.472	0.3199	0.438	0.4557
Ring Empty	Ring ssDNA	a1	2500	0.335	0.5385	0.542	0.6227
Ring Empty	Ring ssDNA	a1	5000	0.266	0.5827	0.452	0.5613
Ring Empty	Ring ssDNA	a1	10000	0.440	0.4679	0.456	0.5741
Ring Empty	Ring ssDNA	a1	25000	0.320	0.3521	0.425	0.3715
Ring Empty	Ring ssDNA	a1	50000	0.355	0.5869	0.484	0.8508
Ring Empty	Ring ssDNA	a1	100000	0.311	0.6401	0.505	0.9606
Ring Empty	Ring ssDNA	a1	Total	0.227	0.2623	0.465	0.2262
Ring Empty	Ring ssDNA	a2	500	2.021	0.0006	0.744	0.0032
Ring Empty	Ring ssDNA	a2	1000	0.298	0.5783	0.561	0.4683
Ring Empty	Ring ssDNA	a2	2500	0.703	0.1914	0.425	0.3805
Ring Empty	Ring ssDNA	a2	5000	0.585	0.3333	0.562	0.4645
Ring Empty	Ring ssDNA	a2	10000	0.221	0.6069	0.557	0.4685
Ring Empty	Ring ssDNA	a2	25000	0.140	0.6527	0.560	0.4697
Ring Empty	Ring ssDNA	a2	50000	0.160	0.7796	0.513	0.8694
Ring Empty	Ring ssDNA	a2	100000	0.296	0.5241	0.444	0.5683
Ring Empty	Ring ssDNA	a2	Total	0.301	0.0948	0.537	0.2140

Ring Empty	Ring ssDNA	b1	500	3.219	0.0002	0.924	0.0002
Ring Empty	Ring ssDNA	b1	1000	2.669	0.0004	0.761	0.0016
Ring Empty	Ring ssDNA	b1	2500	0.871	0.0434	0.786	0.0006
Ring Empty	Ring ssDNA	b1	5000	0.556	0.0088	0.553	0.5361
Ring Empty	Ring ssDNA	b1	10000	0.284	0.4533	0.620	0.1388
Ring Empty	Ring ssDNA	b1	25000	0.551	0.1064	0.644	0.0888
Ring Empty	Ring ssDNA	b1	50000	0.594	0.2418	0.333	0.0304
Ring Empty	Ring ssDNA	b1	100000	1.387	0.0186	0.876	0.0002
Ring Empty	Ring ssDNA	b1	Total	0.735	0.0216	0.608	0.0006
Ring Empty	Ring ssDNA	c1	500	4.738	0.0002	0.838	0.0002
Ring Empty	Ring ssDNA	c1	1000	2.950	0.0002	0.847	0.0002
Ring Empty	Ring ssDNA	c1	2500	2.549	0.0002	0.863	0.0002
Ring Empty	Ring ssDNA	c1	5000	3.570	0.0002	0.966	0.0002
Ring Empty	Ring ssDNA	c1	10000	0.253	0.5375	0.604	0.1986
Ring Empty	Ring ssDNA	c1	25000	0.138	0.5705	0.424	0.3513
Ring Empty	Ring ssDNA	c1	50000	0.403	0.5297	0.477	0.7734
Ring Empty	Ring ssDNA	c1	100000	0.604	0.1856	0.482	0.8530
Ring Empty	Ring ssDNA	c1	Total	0.788	0.0002	0.631	0.0002
Ring Empty	Ring ssDNA	d1	500	0.598	0.3035	0.377	0.1412
Ring Empty	Ring ssDNA	d1	1000	1.195	0.0042	0.245	0.0008
Ring Empty	Ring ssDNA	d1	2500	1.487	0.0008	0.248	0.0014
Ring Empty	Ring ssDNA	d1	5000	2.774	0.0002	0.138	0.0002
Ring Empty	Ring ssDNA	d1	10000	1.217	0.0046	0.303	0.0156
Ring Empty	Ring ssDNA	d1	25000	0.885	0.0188	0.324	0.0320
Ring Empty	Ring ssDNA	d1	50000	0.003	0.8534	0.414	0.2895

Ring Empty	Ring ssDNA	d1	100000	2.085	0.0032	0.737	0.0098
Ring Empty	Ring ssDNA	d1	Total	0.288	0.1900	0.445	0.0608
Ring Empty	Ring ssDNA	e1	500	6.852	0.0002	0.032	0.0002
Ring Empty	Ring ssDNA	e1	1000	5.723	0.0002	0.003	0.0002
Ring Empty	Ring ssDNA	e1	2500	3.848	0.0002	0.026	0.0002
Ring Empty	Ring ssDNA	e1	5000	2.474	0.0002	0.202	0.0006
Ring Empty	Ring ssDNA	e1	10000	1.521	0.0020	0.275	0.0042
Ring Empty	Ring ssDNA	e1	25000	0.280	0.6397	0.578	0.3497
Ring Empty	Ring ssDNA	e1	50000	0.116	0.7790	0.662	0.0444
Ring Empty	Ring ssDNA	e1	100000	1.358	0.0170	0.338	0.0888
Ring Empty	Ring ssDNA	e1	Total	0.925	0.0002	0.366	0.0002
Ring Empty	Ring ssDNA	PhaseDelay	500	0.521	0.3021	0.420	0.3495
Ring Empty	Ring ssDNA	PhaseDelay	1000	0.658	0.1510	0.397	0.2168
Ring Empty	Ring ssDNA	PhaseDelay	2500	1.460	0.0020	0.729	0.0052
Ring Empty	Ring ssDNA	PhaseDelay	5000	0.586	0.3647	0.408	0.2757
Ring Empty	Ring ssDNA	PhaseDelay	10000	0.009	0.8930	0.454	0.5531
Ring Empty	Ring ssDNA	PhaseDelay	25000	1.000	0.1530	0.638	0.0978
Ring Empty	Ring ssDNA	PhaseDelay	50000	0.000	0.3813	0.483	0.8176
Ring Empty	Ring ssDNA	PhaseDelay	100000	0.000	0.9560	0.497	0.9802
Ring Empty	Ring ssDNA	PhaseDelay	Total	0.064	0.3793	0.504	0.9006
Ring Empty	Ring scDNA	a1	500	0.476	0.2957	0.481	0.8730
Ring Empty	Ring scDNA	a1	1000	0.336	0.5265	0.417	0.4395
Ring Empty	Ring scDNA	a1	2500	0.676	0.2845	0.548	0.6421
Ring Empty	Ring scDNA	a1	5000	0.325	0.5971	0.437	0.5591
Ring Empty	Ring scDNA	a1	10000	0.408	0.6713	0.383	0.2847

Ring Empty	Ring scDNA	a1	25000	0.749	0.4121	0.620	0.2591
Ring Empty	Ring scDNA	a1	50000	0.118	0.8318	0.411	0.4061
Ring Empty	Ring scDNA	a1	100000	0.842	0.2020	0.626	0.2749
Ring Empty	Ring scDNA	a1	Total	0.076	0.6953	0.478	0.5577
Ring Empty	Ring scDNA	a2	500	0.703	0.2388	0.587	0.4153
Ring Empty	Ring scDNA	a2	1000	0.505	0.6091	0.583	0.4215
Ring Empty	Ring scDNA	a2	2500	0.465	0.5553	0.405	0.3567
Ring Empty	Ring scDNA	a2	5000	0.039	0.9288	0.512	0.9254
Ring Empty	Ring scDNA	a2	10000	2.003	0.0238	0.644	0.1816
Ring Empty	Ring scDNA	a2	25000	0.988	0.1462	0.337	0.1164
Ring Empty	Ring scDNA	a2	50000	1.159	0.0408	0.696	0.0518
Ring Empty	Ring scDNA	a2	100000	0.816	0.3595	0.394	0.3651
Ring Empty	Ring scDNA	a2	Total	0.430	0.1186	0.516	0.6461
Ring Empty	Ring scDNA	b1	500	1.864	0.0402	0.678	0.0892
Ring Empty	Ring scDNA	b1	1000	2.107	0.0108	0.686	0.0792
Ring Empty	Ring scDNA	b1	2500	0.009	0.9898	0.615	0.2501
Ring Empty	Ring scDNA	b1	5000	1.812	0.0258	0.853	0.0008
Ring Empty	Ring scDNA	b1	10000	6.482	0.0002	1.000	0.0002
Ring Empty	Ring scDNA	b1	25000	1.973	0.0008	0.841	0.0014
Ring Empty	Ring scDNA	b1	50000	0.120	0.6379	0.431	0.5095
Ring Empty	Ring scDNA	b1	100000	1.599	0.0900	0.227	0.0168
Ring Empty	Ring scDNA	b1	Total	0.821	0.0024	0.585	0.0250
Ring Empty	Ring scDNA	c1	500	3.158	0.0028	0.250	0.0178
Ring Empty	Ring scDNA	c1	1000	2.113	0.0012	0.182	0.0020
Ring Empty	Ring scDNA	c1	2500	2.519	0.0008	0.087	0.0002

Ring Empty	Ring scDNA	c1	5000	2.958	0.0002	0.143	0.0006
Ring Empty	Ring scDNA	c1	10000	0.343	0.4297	0.492	0.9588
Ring Empty	Ring scDNA	c1	25000	0.173	0.8344	0.580	0.4605
Ring Empty	Ring scDNA	c1	50000	0.898	0.2190	0.268	0.0210
Ring Empty	Ring scDNA	c1	100000	0.062	0.9070	0.591	0.4245
Ring Empty	Ring scDNA	c1	Total	0.772	0.0006	0.397	0.0040
Ring Empty	Ring scDNA	d1	500	0.128	0.8268	0.572	0.5067
Ring Empty	Ring scDNA	d1	1000	0.603	0.2322	0.583	0.4307
Ring Empty	Ring scDNA	d1	2500	3.609	0.0042	0.843	0.0004
Ring Empty	Ring scDNA	d1	5000	4.664	0.0038	0.873	0.0004
Ring Empty	Ring scDNA	d1	10000	4.223	0.0002	0.886	0.0004
Ring Empty	Ring scDNA	d1	25000	0.313	0.5563	0.547	0.6557
Ring Empty	Ring scDNA	d1	50000	1.244	0.0526	0.341	0.1224
Ring Empty	Ring scDNA	d1	100000	0.400	0.6933	0.601	0.3769
Ring Empty	Ring scDNA	d1	Total	0.020	0.9262	0.518	0.6171
Ring Empty	Ring scDNA	e1	500	12.865	0.0002	1.000	0.0002
Ring Empty	Ring scDNA	e1	1000	12.217	0.0002	1.000	0.0002
Ring Empty	Ring scDNA	e1	2500	7.666	0.0002	0.967	0.0002
Ring Empty	Ring scDNA	e1	5000	4.282	0.0002	0.944	0.0002
Ring Empty	Ring scDNA	e1	10000	2.013	0.0064	0.826	0.0012
Ring Empty	Ring scDNA	e1	25000	0.391	0.3841	0.605	0.3197
Ring Empty	Ring scDNA	e1	50000	0.788	0.0910	0.746	0.0150
Ring Empty	Ring scDNA	e1	100000	0.245	0.7047	0.586	0.4681
Ring Empty	Ring scDNA	e1	Total	1.908	0.0002	0.740	0.0002
Ring Empty	Ring scDNA	PhaseDelay	500	0.171	0.8872	0.591	0.4043

Ring Empty	Ring scDNA	PhaseDelay	1000	1.259	0.0622	0.258	0.0208
Ring Empty	Ring scDNA	PhaseDelay	2500	1.007	0.0762	0.314	0.0674
Ring Empty	Ring scDNA	PhaseDelay	5000	0.527	0.3175	0.351	0.1606
Ring Empty	Ring scDNA	PhaseDelay	10000	0.014	0.6903	0.464	0.7534
Ring Empty	Ring scDNA	PhaseDelay	25000	0.000	0.5119	0.549	0.6355
Ring Empty	Ring scDNA	PhaseDelay	50000	0.000	1.0000	0.497	0.9822
Ring Empty	Ring scDNA	PhaseDelay	100000	0.000	1.0000	0.449	0.6747
Ring Empty	Ring scDNA	PhaseDelay	Total	0.000	1.0000	0.489	0.7620
Ring ssDNA	Ring scDNA	a1	500	3.330	0.0002	0.851	0.0004
Ring ssDNA	Ring scDNA	a1	1000	0.158	0.7500	0.500	1.0000
Ring ssDNA	Ring scDNA	a1	2500	0.443	0.3773	0.558	0.5701
Ring ssDNA	Ring scDNA	a1	5000	0.038	0.9326	0.483	0.8814
Ring ssDNA	Ring scDNA	a1	10000	0.061	0.9244	0.409	0.3857
Ring ssDNA	Ring scDNA	a1	25000	0.996	0.0854	0.679	0.0858
Ring ssDNA	Ring scDNA	a1	50000	0.257	0.6607	0.429	0.4541
Ring ssDNA	Ring scDNA	a1	100000	2.259	0.0012	0.711	0.0498
Ring ssDNA	Ring scDNA	a1	Total	0.145	0.5851	0.513	0.7051
Ring ssDNA	Ring scDNA	a2	500	2.559	0.0002	0.128	0.0006
Ring ssDNA	Ring scDNA	a2	1000	0.385	0.6329	0.517	0.8682
Ring ssDNA	Ring scDNA	a2	2500	0.079	0.8076	0.451	0.6237
Ring ssDNA	Ring scDNA	a2	5000	0.846	0.2264	0.408	0.3633
Ring ssDNA	Ring scDNA	a2	10000	1.748	0.0136	0.599	0.3353
Ring ssDNA	Ring scDNA	a2	25000	0.996	0.1042	0.278	0.0284
Ring ssDNA	Ring scDNA	a2	50000	1.444	0.0154	0.681	0.0590
Ring ssDNA	Ring scDNA	a2	100000	0.978	0.0964	0.401	0.3743

Ring ssDNA	Ring scDNA	a2	Total	0.170	0.3669	0.485	0.6637
Ring ssDNA	Ring scDNA	b1	500	1.442	0.0184	0.170	0.0008
Ring ssDNA	Ring scDNA	b1	1000	0.569	0.2683	0.328	0.0952
Ring ssDNA	Ring scDNA	b1	2500	1.093	0.0526	0.247	0.0106
Ring ssDNA	Ring scDNA	b1	5000	8.938	0.0002	0.928	0.0002
Ring ssDNA	Ring scDNA	b1	10000	14.360	0.0002	0.946	0.0002
Ring ssDNA	Ring scDNA	b1	25000	2.030	0.0004	0.756	0.0082
Ring ssDNA	Ring scDNA	b1	50000	0.824	0.2509	0.541	0.6935
Ring ssDNA	Ring scDNA	b1	100000	7.666	0.0008	0.012	0.0002
Ring ssDNA	Ring scDNA	b1	Total	0.062	0.5461	0.456	0.2100
Ring ssDNA	Ring scDNA	c1	500	6.890	0.0004	0.098	0.0002
Ring ssDNA	Ring scDNA	c1	1000	13.865	0.0002	0.000	0.0002
Ring ssDNA	Ring scDNA	c1	2500	5.602	0.0002	0.000	0.0002
Ring ssDNA	Ring scDNA	c1	5000	5.906	0.0002	0.000	0.0002
Ring ssDNA	Ring scDNA	c1	10000	1.404	0.0422	0.318	0.0746
Ring ssDNA	Ring scDNA	c1	25000	0.449	0.2306	0.636	0.1842
Ring ssDNA	Ring scDNA	c1	50000	0.763	0.1672	0.244	0.0060
Ring ssDNA	Ring scDNA	c1	100000	0.580	0.2857	0.591	0.4195
Ring ssDNA	Ring scDNA	c1	Total	1.529	0.0002	0.300	0.0002
Ring ssDNA	Ring scDNA	d1	500	0.817	0.1398	0.682	0.0688
Ring ssDNA	Ring scDNA	d1	1000	3.180	0.0002	0.853	0.0006
Ring ssDNA	Ring scDNA	d1	2500	4.179	0.0002	0.948	0.0002
Ring ssDNA	Ring scDNA	d1	5000	8.595	0.0002	0.991	0.0002
Ring ssDNA	Ring scDNA	d1	10000	6.487	0.0002	0.983	0.0002
Ring ssDNA	Ring scDNA	d1	25000	2.708	0.0002	0.818	0.0006

Ring ssDNA	Ring scDNA	d1	50000	1.129	0.1218	0.413	0.3691
Ring ssDNA	Ring scDNA	d1	100000	7.192	0.0002	0.215	0.0078
Ring ssDNA	Ring scDNA	d1	Total	0.290	0.2571	0.576	0.0356
Ring ssDNA	Ring scDNA	e1	500	29.847	0.0002	1.000	0.0002
Ring ssDNA	Ring scDNA	e1	1000	37.807	0.0002	1.000	0.0002
Ring ssDNA	Ring scDNA	e1	2500	12.429	0.0002	1.000	0.0002
Ring ssDNA	Ring scDNA	e1	5000	8.150	0.0002	1.000	0.0002
Ring ssDNA	Ring scDNA	e1	10000	4.694	0.0002	0.949	0.0002
Ring ssDNA	Ring scDNA	e1	25000	1.725	0.0030	0.642	0.1658
Ring ssDNA	Ring scDNA	e1	50000	0.888	0.1790	0.585	0.3661
Ring ssDNA	Ring scDNA	e1	100000	1.155	0.0370	0.649	0.1758
Ring ssDNA	Ring scDNA	e1	Total	3.235	0.0002	0.801	0.0002
Ring ssDNA	Ring scDNA	PhaseDelay	500	0.421	0.2747	0.643	0.1614
Ring ssDNA	Ring scDNA	PhaseDelay	1000	0.581	0.4729	0.378	0.2194
Ring ssDNA	Ring scDNA	PhaseDelay	2500	2.432	0.0004	0.203	0.0012
Ring ssDNA	Ring scDNA	PhaseDelay	5000	0.022	0.9958	0.440	0.5533
Ring ssDNA	Ring scDNA	PhaseDelay	10000	0.023	0.3821	0.480	0.8560
Ring ssDNA	Ring scDNA	PhaseDelay	25000	1.392	0.1612	0.377	0.2212
Ring ssDNA	Ring scDNA	PhaseDelay	50000	0.000	0.4293	0.537	0.7013
Ring ssDNA	Ring scDNA	PhaseDelay	100000	0.112	0.6489	0.457	0.7033
Ring ssDNA	Ring scDNA	PhaseDelay	Total	0.061	0.7570	0.488	0.7355
Wire Empty	Wire ssDNA	a1	500	0.845	0.2008	0.337	0.0638
Wire Empty	Wire ssDNA	a1	1000	0.560	0.3361	0.399	0.3133
Wire Empty	Wire ssDNA	a1	2500	0.223	0.7910	0.517	0.8474
Wire Empty	Wire ssDNA	a1	5000	0.979	0.1252	0.395	0.2657

Wire Empty	Wire ssDNA	a1	10000	1.748	0.0082	0.732	0.0100
Wire Empty	Wire ssDNA	a1	25000	0.475	0.4795	0.427	0.4433
Wire Empty	Wire ssDNA	a1	50000	0.424	0.4945	0.611	0.2144
Wire Empty	Wire ssDNA	a1	100000	0.046	0.9822	0.444	0.5927
Wire Empty	Wire ssDNA	a1	Total	0.005	0.9512	0.515	0.6519
Wire Empty	Wire ssDNA	a2	500	0.756	0.0784	0.652	0.0856
Wire Empty	Wire ssDNA	a2	1000	0.138	0.6327	0.508	0.9494
Wire Empty	Wire ssDNA	a2	2500	0.004	1.0000	0.556	0.5377
Wire Empty	Wire ssDNA	a2	5000	0.411	0.4769	0.561	0.5297
Wire Empty	Wire ssDNA	a2	10000	2.466	0.0008	0.256	0.0070
Wire Empty	Wire ssDNA	a2	25000	0.340	0.9864	0.573	0.4371
Wire Empty	Wire ssDNA	a2	50000	0.120	0.8188	0.462	0.6925
Wire Empty	Wire ssDNA	a2	100000	0.463	0.3953	0.604	0.3109
Wire Empty	Wire ssDNA	a2	Total	0.258	0.4927	0.490	0.7664
Wire Empty	Wire ssDNA	b1	500	0.134	0.7562	0.466	0.7145
Wire Empty	Wire ssDNA	b1	1000	0.537	0.7115	0.505	0.9618
Wire Empty	Wire ssDNA	b1	2500	0.680	0.1036	0.598	0.2795
Wire Empty	Wire ssDNA	b1	5000	1.640	0.0232	0.361	0.1398
Wire Empty	Wire ssDNA	b1	10000	0.068	0.7229	0.505	0.9668
Wire Empty	Wire ssDNA	b1	25000	0.233	0.8094	0.551	0.6059
Wire Empty	Wire ssDNA	b1	50000	0.666	0.0716	0.621	0.1754
Wire Empty	Wire ssDNA	b1	100000	0.530	0.1964	0.607	0.3113
Wire Empty	Wire ssDNA	b1	Total	0.064	0.7832	0.512	0.7053
Wire Empty	Wire ssDNA	c1	500	0.383	0.3223	0.605	0.2501
Wire Empty	Wire ssDNA	c1	1000	0.283	0.6855	0.576	0.4493

Wire Empty	Wire ssDNA	c1	2500	1.539	0.0212	0.708	0.0204
Wire Empty	Wire ssDNA	c1	5000	0.117	0.7604	0.489	0.9240
Wire Empty	Wire ssDNA	c1	10000	0.305	0.4721	0.568	0.4683
Wire Empty	Wire ssDNA	c1	25000	1.415	0.0864	0.651	0.1156
Wire Empty	Wire ssDNA	c1	50000	1.490	0.0336	0.717	0.0114
Wire Empty	Wire ssDNA	c1	100000	1.967	0.0038	0.726	0.0266
Wire Empty	Wire ssDNA	c1	Total	0.432	0.0002	0.635	0.0002
Wire Empty	Wire ssDNA	d1	500	0.026	0.8002	0.501	1.0000
Wire Empty	Wire ssDNA	d1	1000	0.278	0.4521	0.443	0.5703
Wire Empty	Wire ssDNA	d1	2500	0.749	0.2581	0.598	0.2785
Wire Empty	Wire ssDNA	d1	5000	2.456	0.0002	0.811	0.0008
Wire Empty	Wire ssDNA	d1	10000	0.866	0.1254	0.618	0.2148
Wire Empty	Wire ssDNA	d1	25000	0.518	0.3567	0.582	0.4099
Wire Empty	Wire ssDNA	d1	50000	1.779	0.0064	0.709	0.0130
Wire Empty	Wire ssDNA	d1	100000	1.951	0.0064	0.770	0.0050
Wire Empty	Wire ssDNA	d1	Total	0.348	0.1056	0.587	0.0058
Wire Empty	Wire ssDNA	e1	500	1.457	0.0068	0.760	0.0028
Wire Empty	Wire ssDNA	e1	1000	0.936	0.1332	0.650	0.1288
Wire Empty	Wire ssDNA	e1	2500	1.081	0.0244	0.746	0.0058
Wire Empty	Wire ssDNA	e1	5000	1.647	0.0048	0.213	0.0014
Wire Empty	Wire ssDNA	e1	10000	0.074	0.8264	0.522	0.8152
Wire Empty	Wire ssDNA	e1	25000	0.286	0.7017	0.496	0.9640
Wire Empty	Wire ssDNA	e1	50000	0.419	0.2372	0.379	0.1668
Wire Empty	Wire ssDNA	e1	100000	1.101	0.0678	0.307	0.0588
Wire Empty	Wire ssDNA	e1	Total	0.156	0.4213	0.482	0.5881

Wire Empty	Wire ssDNA	PhaseDelay	500	1.063	0.1030	0.408	0.2999
Wire Empty	Wire ssDNA	PhaseDelay	1000	0.172	0.9610	0.502	1.0000
Wire Empty	Wire ssDNA	PhaseDelay	2500	0.104	0.9014	0.523	0.8026
Wire Empty	Wire ssDNA	PhaseDelay	5000	0.028	0.8484	0.487	0.8930
Wire Empty	Wire ssDNA	PhaseDelay	10000	0.618	0.3965	0.429	0.4451
Wire Empty	Wire ssDNA	PhaseDelay	25000	0.748	0.6151	0.454	0.6399
Wire Empty	Wire ssDNA	PhaseDelay	50000	0.312	0.4083	0.596	0.2735
Wire Empty	Wire ssDNA	PhaseDelay	100000	0.000	0.5529	0.554	0.6187
Wire Empty	Wire ssDNA	PhaseDelay	Total	0.040	0.7500	0.503	0.9308
Wire Empty	Wire scDNA	a1	500	3.465	0.0002	0.089	0.0002
Wire Empty	Wire scDNA	a1	1000	1.215	0.0562	0.314	0.0402
Wire Empty	Wire scDNA	a1	2500	0.245	0.7055	0.413	0.3037
Wire Empty	Wire scDNA	a1	5000	0.736	0.1680	0.619	0.1980
Wire Empty	Wire scDNA	a1	10000	0.044	0.9294	0.477	0.7886
Wire Empty	Wire scDNA	a1	25000	1.050	0.0642	0.352	0.1076
Wire Empty	Wire scDNA	a1	50000	0.634	0.2014	0.407	0.2965
Wire Empty	Wire scDNA	a1	100000	4.265	0.0002	0.097	0.0002
Wire Empty	Wire scDNA	a1	Total	0.509	0.0312	0.435	0.0370
Wire Empty	Wire scDNA	a2	500	2.991	0.0002	0.870	0.0002
Wire Empty	Wire scDNA	a2	1000	0.938	0.1270	0.673	0.0564
Wire Empty	Wire scDNA	a2	2500	0.228	0.6205	0.590	0.2915
Wire Empty	Wire scDNA	a2	5000	0.355	0.2687	0.413	0.3317
Wire Empty	Wire scDNA	a2	10000	0.428	0.6561	0.464	0.6763
Wire Empty	Wire scDNA	a2	25000	0.756	0.3213	0.570	0.4385
Wire Empty	Wire scDNA	a2	50000	0.287	0.6161	0.488	0.8972

Wire Empty	Wire scDNA	a2	100000	1.933	0.0006	0.816	0.0008
Wire Empty	Wire scDNA	a2	Total	0.285	0.1134	0.552	0.0876
Wire Empty	Wire scDNA	b1	500	0.952	0.1880	0.643	0.0930
Wire Empty	Wire scDNA	b1	1000	0.409	0.4771	0.622	0.1704
Wire Empty	Wire scDNA	b1	2500	1.036	0.0432	0.676	0.0316
Wire Empty	Wire scDNA	b1	5000	0.090	0.9458	0.493	0.9472
Wire Empty	Wire scDNA	b1	10000	0.534	0.3913	0.586	0.3087
Wire Empty	Wire scDNA	b1	25000	2.201	0.0008	0.270	0.0110
Wire Empty	Wire scDNA	b1	50000	1.816	0.0012	0.140	0.0002
Wire Empty	Wire scDNA	b1	100000	3.507	0.0002	0.814	0.0006
Wire Empty	Wire scDNA	b1	Total	0.180	0.5793	0.520	0.5199
Wire Empty	Wire scDNA	c1	500	0.430	0.3959	0.431	0.4273
Wire Empty	Wire scDNA	c1	1000	0.174	0.6359	0.410	0.3447
Wire Empty	Wire scDNA	c1	2500	0.676	0.1168	0.335	0.0452
Wire Empty	Wire scDNA	c1	5000	0.248	0.9078	0.459	0.6559
Wire Empty	Wire scDNA	c1	10000	0.338	0.4833	0.443	0.5083
Wire Empty	Wire scDNA	c1	25000	0.720	0.1084	0.634	0.1408
Wire Empty	Wire scDNA	c1	50000	1.430	0.0614	0.682	0.0368
Wire Empty	Wire scDNA	c1	100000	0.690	0.2817	0.657	0.0820
Wire Empty	Wire scDNA	c1	Total	0.017	0.9130	0.529	0.3413
Wire Empty	Wire scDNA	d1	500	0.205	0.6225	0.365	0.1230
Wire Empty	Wire scDNA	d1	1000	0.597	0.2140	0.389	0.2290
Wire Empty	Wire scDNA	d1	2500	0.954	0.0386	0.357	0.0832
Wire Empty	Wire scDNA	d1	5000	0.968	0.0534	0.377	0.1642
Wire Empty	Wire scDNA	d1	10000	1.414	0.0070	0.330	0.0422

Wire Empty	Wire scDNA	d1	25000	1.703	0.0062	0.650	0.0966
Wire Empty	Wire scDNA	d1	50000	0.682	0.0972	0.634	0.1230
Wire Empty	Wire scDNA	d1	100000	3.245	0.0002	0.853	0.0002
Wire Empty	Wire scDNA	d1	Total	0.340	0.1800	0.538	0.2068
Wire Empty	Wire scDNA	e1	500	0.169	0.7319	0.539	0.6469
Wire Empty	Wire scDNA	e1	1000	0.913	0.0774	0.339	0.0828
Wire Empty	Wire scDNA	e1	2500	1.257	0.0178	0.316	0.0266
Wire Empty	Wire scDNA	e1	5000	1.152	0.0444	0.316	0.0388
Wire Empty	Wire scDNA	e1	10000	0.135	0.8228	0.563	0.4603
Wire Empty	Wire scDNA	e1	25000	4.026	0.0002	0.735	0.0080
Wire Empty	Wire scDNA	e1	50000	0.554	0.3203	0.593	0.2895
Wire Empty	Wire scDNA	e1	100000	0.886	0.0278	0.336	0.0700
Wire Empty	Wire scDNA	e1	Total	0.321	0.0320	0.466	0.2783
Wire Empty	Wire scDNA	PhaseDelay	500	0.297	0.7594	0.515	0.8808
Wire Empty	Wire scDNA	PhaseDelay	1000	0.149	0.5611	0.584	0.3677
Wire Empty	Wire scDNA	PhaseDelay	2500	0.480	0.4199	0.620	0.1440
Wire Empty	Wire scDNA	PhaseDelay	5000	0.575	0.3833	0.598	0.2817
Wire Empty	Wire scDNA	PhaseDelay	10000	0.734	0.4835	0.473	0.7522
Wire Empty	Wire scDNA	PhaseDelay	25000	0.000	1.0000	0.485	0.8722
Wire Empty	Wire scDNA	PhaseDelay	50000	1.414	0.1318	0.463	0.6819
Wire Empty	Wire scDNA	PhaseDelay	100000	0.000	0.7800	0.575	0.4205
Wire Empty	Wire scDNA	PhaseDelay	Total	0.207	0.1888	0.526	0.3859
Wire ssDNA	Wire scDNA	a1	500	2.042	0.0010	0.183	0.0002
Wire ssDNA	Wire scDNA	a1	1000	0.443	0.2454	0.404	0.3075
Wire ssDNA	Wire scDNA	a1	2500	0.382	0.2905	0.427	0.4051

Wire ssDNA	Wire scDNA	a1	5000	1.391	0.0376	0.642	0.1042
Wire ssDNA	Wire scDNA	a1	10000	1.537	0.0052	0.296	0.0238
Wire ssDNA	Wire scDNA	a1	25000	0.830	0.1506	0.410	0.3323
Wire ssDNA	Wire scDNA	a1	50000	0.783	0.1160	0.335	0.0606
Wire ssDNA	Wire scDNA	a1	100000	2.517	0.0002	0.206	0.0010
Wire ssDNA	Wire scDNA	a1	Total	0.463	0.0234	0.430	0.0228
Wire ssDNA	Wire scDNA	a2	500	2.118	0.0006	0.800	0.0002
Wire ssDNA	Wire scDNA	a2	1000	0.745	0.1680	0.660	0.0934
Wire ssDNA	Wire scDNA	a2	2500	0.168	0.7926	0.513	0.8794
Wire ssDNA	Wire scDNA	a2	5000	0.600	0.2324	0.412	0.3139
Wire ssDNA	Wire scDNA	a2	10000	1.776	0.0038	0.684	0.0436
Wire ssDNA	Wire scDNA	a2	25000	0.602	0.3451	0.529	0.7658
Wire ssDNA	Wire scDNA	a2	50000	0.138	0.7514	0.531	0.7377
Wire ssDNA	Wire scDNA	a2	100000	0.761	0.2114	0.652	0.1276
Wire ssDNA	Wire scDNA	a2	Total	0.466	0.0576	0.555	0.0832
Wire ssDNA	Wire scDNA	b1	500	0.668	0.1750	0.667	0.0534
Wire ssDNA	Wire scDNA	b1	1000	1.026	0.2517	0.634	0.1460
Wire ssDNA	Wire scDNA	b1	2500	0.385	0.4303	0.585	0.3327
Wire ssDNA	Wire scDNA	b1	5000	1.860	0.0018	0.674	0.0488
Wire ssDNA	Wire scDNA	b1	10000	0.599	0.3117	0.571	0.4509
Wire ssDNA	Wire scDNA	b1	25000	2.084	0.0010	0.268	0.0146
Wire ssDNA	Wire scDNA	b1	50000	2.069	0.0002	0.140	0.0002
Wire ssDNA	Wire scDNA	b1	100000	0.786	0.0760	0.600	0.3191
Wire ssDNA	Wire scDNA	b1	Total	0.126	0.6389	0.510	0.7469
Wire ssDNA	Wire scDNA	c1	500	0.698	0.1302	0.366	0.1230

Wire ssDNA	Wire scDNA	c1	1000	0.228	0.4765	0.373	0.1814
Wire ssDNA	Wire scDNA	c1	2500	2.304	0.0002	0.160	0.0002
Wire ssDNA	Wire scDNA	c1	5000	0.125	0.7668	0.472	0.7586
Wire ssDNA	Wire scDNA	c1	10000	0.632	0.1894	0.371	0.1630
Wire ssDNA	Wire scDNA	c1	25000	1.081	0.1856	0.444	0.5489
Wire ssDNA	Wire scDNA	c1	50000	0.188	0.8764	0.450	0.5813
Wire ssDNA	Wire scDNA	c1	100000	1.885	0.0052	0.267	0.0176
Wire ssDNA	Wire scDNA	c1	Total	0.515	0.0002	0.384	0.0006
Wire ssDNA	Wire scDNA	d1	500	0.247	0.5747	0.381	0.1832
Wire ssDNA	Wire scDNA	d1	1000	0.255	0.4769	0.442	0.5475
Wire ssDNA	Wire scDNA	d1	2500	1.518	0.0032	0.288	0.0112
Wire ssDNA	Wire scDNA	d1	5000	2.974	0.0004	0.192	0.0004
Wire ssDNA	Wire scDNA	d1	10000	1.562	0.0040	0.249	0.0054
Wire ssDNA	Wire scDNA	d1	25000	0.387	0.2757	0.524	0.8128
Wire ssDNA	Wire scDNA	d1	50000	1.249	0.1476	0.333	0.0616
Wire ssDNA	Wire scDNA	d1	100000	0.370	0.4341	0.478	0.8370
Wire ssDNA	Wire scDNA	d1	Total	0.018	0.8792	0.453	0.1404
Wire ssDNA	Wire scDNA	e1	500	1.370	0.0088	0.259	0.0040
Wire ssDNA	Wire scDNA	e1	1000	1.434	0.0150	0.297	0.0300
Wire ssDNA	Wire scDNA	e1	2500	2.023	0.0008	0.140	0.0002
Wire ssDNA	Wire scDNA	e1	5000	0.454	0.5191	0.590	0.3113
Wire ssDNA	Wire scDNA	e1	10000	0.033	0.9700	0.516	0.8798
Wire ssDNA	Wire scDNA	e1	25000	2.906	0.0002	0.737	0.0068
Wire ssDNA	Wire scDNA	e1	50000	1.219	0.0184	0.758	0.0046
Wire ssDNA	Wire scDNA	e1	100000	0.553	0.2400	0.577	0.4375

Wire ssDNA	Wire scDNA	e1	Total	0.069	0.7113	0.504	0.8984
Wire ssDNA	Wire scDNA	PhaseDelay	500	0.789	0.1362	0.630	0.1326
Wire ssDNA	Wire scDNA	PhaseDelay	1000	0.321	0.7694	0.568	0.4707
Wire ssDNA	Wire scDNA	PhaseDelay	2500	0.329	0.6215	0.614	0.1904
Wire ssDNA	Wire scDNA	PhaseDelay	5000	0.807	0.1892	0.635	0.1244
Wire ssDNA	Wire scDNA	PhaseDelay	10000	0.106	0.5789	0.559	0.5299
Wire ssDNA	Wire scDNA	PhaseDelay	25000	0.798	0.3487	0.555	0.5487
Wire ssDNA	Wire scDNA	PhaseDelay	50000	1.414	0.2623	0.351	0.0988
Wire ssDNA	Wire scDNA	PhaseDelay	100000	0.000	1.0000	0.491	0.9302
Wire ssDNA	Wire scDNA	PhaseDelay	Total	0.245	0.1348	0.526	0.4079
